# Anti-Inflammatory and Chondroprotective Effects of Vanillic Acid and Epimedin C in Human Osteoarthritic Chondrocytes

**DOI:** 10.3390/biom10060932

**Published:** 2020-06-19

**Authors:** Reihane Ziadlou, Andrea Barbero, Ivan Martin, Xinluan Wang, Ling Qin, Mauro Alini, Sibylle Grad

**Affiliations:** 1 AO Research Institute Davos, 7270 Davos Platz, Switzerland; reihane.ziadlou@aofoundation.org (R.Z.); mauro.alini@aofoundation.org (M.A.); 2 Department of Biomedical Engineering, University of Basel, 4123 Allschwil, Switzerland; ivan.martin@usb.ch; 3 Department of Biomedicine, University Hospital Basel, University of Basel, 4001 Basel, Switzerland; andrea.barbero@usb.ch; 4 Translational Medicine R&D Center, Shenzhen Institutes of Advanced Technology, Chinese Academy of Sciences, Shenzhen 518057, China; xl.wang@siat.ac.cn (X.W.); lingqin@cuhk.edu.hk (L.Q.); 5 Department of Orthopaedics & Traumatology, The Chinese University of Hong Kong, Hong Kong SAR, China; 6 Department of Health Sciences and Technology, ETH Zürich, 8092 Zürich, Switzerland

**Keywords:** osteoarthritis, vanillic acid, Epimedin C, anti-inflammatory effects, NF-κB signaling pathway, anabolic, anti-catabolic

## Abstract

In osteoarthritis (OA), inhibition of excessively expressed pro-inflammatory cytokines in the OA joint and increasing the anabolism for cartilage regeneration are necessary. In this ex-vivo study, we used an inflammatory model of human OA chondrocytes microtissues, consisting of treatment with cytokines (interleukin 1β (IL-1β)/tumor necrosis factor α (TNF-α)) with or without supplementation of six herbal compounds with previously identified chondroprotective effect. The compounds were assessed for their capacity to modulate the key catabolic and anabolic factors using several molecular analyses. We selectively investigated the mechanism of action of the two most potent compounds Vanillic acid (VA) and Epimedin C (Epi C). After identification of the anti-inflammatory and anabolic properties of VA and Epi C, the Ingenuity Pathway Analysis showed that in both treatment groups, osteoarthritic signaling pathways were inhibited. In the treatment group with VA, nuclear factor kappa-light-chain-enhancer of activated B cells (NF-κB) signaling was inhibited by attenuation of the nuclear factor of kappa light polypeptide gene enhancer in B-cells inhibitor alpha (IκBα) phosphorylation. Epi C showed a significant anabolic effect by increasing the expression of collagenous and non-collagenous matrix proteins. In conclusion, VA, through inhibition of phosphorylation in NF-κB signaling pathway and Epi C, by increasing the expression of extracellular matrix components, showed significant anti-inflammatory and anabolic properties and might be potentially used in combination to treat or prevent joint OA.

## 1. Introduction

Articular cartilage is an avascular tissue with low cell density that is composed of chondrocytes as the unique cellular component existing in the tissue, and extracellular matrix (ECM) molecules including collagens, proteoglycans, and non-collagen proteins. The matrix turnover rate in the tissue is slow and, in the healthy cartilage, there is a balance between synthesis and degradation of ECM components [1]. In osteoarthritis (OA) this equilibrium is disrupted and the rate of degradation of ECM components is higher than the deposition of newly synthesized molecules [2,3,4]. Previous studies have reported that interleukin 1β (IL1-β) and tumor necrosis factor α (TNF-α) are the main pro-inflammatory cytokines initiating inflammation and causing matrix degradation in OA [5,6]. They activate chondrocytes and synovial cells to produce matrix metalloproteinases (MMPs), aggrecanases, cyclooxygenease-2 (COX-2), prostaglandins (PGEs), and inducible nitric oxide synthase (iNOS) [7]. It is also known that these two cytokines activate or inhibit several different signaling pathways including the nuclear factor kappa-light-chain-enhancer of activated B cells (NF-κB), high-mobility group box 1 (HMGB1), mitogen-activated protein kinases (MAPKs), interleukin-1 receptor (IL-1R)/Toll-like receptor (TLR), and phosphoinositide-3-kinase/protein kinase B/the mammalian target of rapamycin (PI3K/Akt/mTOR) pathways [8,9,10,11,12,13,14,15,16,17,18].

NF-κB controls the expression of genes involved in several physiological responses such as inflammatory response; hence, NF-κB dysregulation can cause inflammatory diseases such as OA and rheumatoid arthritis (RA). The NF-κB proteins normally form a complex with nuclear factor of kappa light polypeptide gene enhancer in B-cells inhibitor alpha (IκBα) protein, which keeps it in an inactive state in the cytoplasm [19]. In the activated canonical pathway, the IκB kinase (IKK) complex phosphorylates IκB proteins, which leads to the activation of NF-κB complex for translocation to the nucleus and the transcription of genes involved in inflammation including immunomodulatory molecules, cytokines, COX-2, MMPs, and iNOS [20,21,22].

Several inhibitors that specifically target signaling pathways by inhibiting key proteins and upstream regulators in the pathway have shown therapeutic potential for treatment of OA in pre-clinical studies [23,24,25,26,27,28,29,30].

Furthermore, IL-1β and TNF-α suppress the expression of the genes associated with the differentiated chondrocyte phenotype, including type II collagen (COL2A1) and aggrecan (ACAN) [31,32]. Presently available drugs for the treatment of OA including nonsteroidal anti-inflammatory drugs (NSAIDs) and COX2 inhibitors are known to have adverse gastrointestinal and cardiovascular effects [31,33]. Moreover, some supplements like glucosamine and chondroitin have been used for treatment of OA, though they showed inconsistent and non-significant effects in the treatment of OA [34,35]. In recent years, several herbal and synthetic small molecules, which were more potent than natural supplements (glucosamine) and could circumvent the side effects of NSAIDs, have been attributed a great potential for OA therapy [36,37,38,39]. Some of these compounds with herbal origins can act through specific signaling pathways and inhibit inflammation or increase matrix synthesis [40,41,42,43]. For instance, compounds including Resveratrol, Curcumin, Honokiol, and Anemonin could block IL-1β-induced NF-κB signaling and inhibit inflammation [44,45,46,47,48]. Furthermore, Berberine and Geniposide could act through inhibition of p38 signaling pathway [49,50]. Icariin, as a widely-studied *herba Epimedii,* has been proposed as a potential promoting herbal molecule for cartilage repair [51,52]. However, there is limited literature available on the anabolic effect of herbal compounds in cartilage regeneration. In our previous study, after screening of 34 most abundant compounds in the herbal Fufang Xian Ling Gu Bao formula (XLGB), which has been used for treatment of osteoporosis, aseptic osteonecrosis, osteoarthritis, and bone fractures in Traditional Chinese Medicine (TCM) [53,54,55,56], we found 6 small molecules with potent anabolic and anti-inflammatory properties [57].

In the current study, RNA sequencing was performed to analyze the transcriptome for gene expression patterns of IL-1β/TNF-α-treated OA chondrocytes in the presence or absence of the six most potent compounds identified in the previous study. These compounds were additionally assayed for their capacity to modulate the transcription and translation of key catabolic and anabolic factors. We then selectively investigated the mechanisms regulating the anti-inflammatory and pro-anabolic effects of the two most effective compounds Vanillic acid (VA) and Epimedin C (Epi C).

## 2. Materials and Methods

### 2.1. Isolation of Human Osteoarthritic Chondrocytes and Cell Expansion

Cartilage tissues were obtained from four patients with end-stage OA after total knee arthroplasty (ages 71, 72, 64, and 82 years; all female) at the University Hospital of Basel under ethical agreement (Ethikkommission beider Basel, Ref.Nr. EK: 78/07). The cells were isolated as described previously elsewhere [58]. In brief, after cutting the tissues with a scalpel into small pieces, they were digested overnight in 0.2% collagenase II (300 U/mg, Worthington Biochemical Corp, Lakewood, NJ, USA) on an orbital shaker at 37 °C. The isolated chondrocytes were expanded for three passages to 80% confluency in basal medium (BM, Dulbecco’s modified Eagle medium, high glucose (DMEM)), 1 mM sodium pyruvate, 10 mM HEPES, 1% penicillin/streptomycin (P/S), and 0.29 mg/mL glutamate (all from Gibco), supplemented with 10% fetal bovine serum (FBS), 1 ng/mL transforming growth factor (TGF)-β1, and 5 ng/mL fibroblast growth factor-2 (FGF-2) (both from Fitzgerald, Acton, MO, USA) in a humidified incubator (37 °C, 5% CO2).

### 2.2. Inflammatory Model of 3D Microtissues for Small Molecules Testing

As previously described [57], chondrocytes 3D microtissues (pellets) were generated after centrifugation of the cells at 400 g for 5 min (2.5 × 10^5^ cells per pellet in 250 μL medium) in v-bottom, non-adherent 96-well plates. 3D microtissues were cultured using standard chondrogenic medium (BM supplemented with 1.25 mg/mL *human* serum albumin (Gibco, Life Technologies Limited, Paisley, UK), ITS-Premix (Corning, Bedford, MA, USA), 0.1 mM ascorbic acid 2-phosphate (Sigma-Aldrich, St. Louis, MO, USA), 1% P/S, 10 ng/mL TGF-β1, and 10^−7^ M dexamethasone (Sigma Aldrich, St. Louis, MO, USA)). After one week of culture in chondrogenic medium for cartilage matrix generation (phase I), pellets were exposed to interleukin 1 beta (IL-1β) and tumor necrosis factor alpha (TNF-α) (both from Peprotech, London, UK), each at 1 ng/mL, for 72 h (phase II) for induction of inflammation. Simultaneously, pellets were treated with the compounds in their effective dosage from our previous screening study [57], including 1 µM of VA, Psoralidin (PS), Protocatechuicaldehyde (PCA), and 25 µM of Epi C, 4-Hydroxybenzoic acid (4-HBA), 5-Hydroxymethylfurfural (5-HMF), or control vehicle (Ctr vehicle) group containing 0.25 % and 0.01% v/v dimethyl sulfoxide (DMSO). At the end of phase II, pellets were harvested for RNA extraction, RNA sequencing, and transcriptional analysis. The remaining pellets were cultured and treated with the compounds for three more days (phase III). At the end of each phase (II and III), the supernatants were collected and stored at −20 °C for further analysis. In phase II and phase III, pellets were cultured in chondropermissive medium (chondrogenic medium deprived of TGF-β1 and dexamethasone). 3D microtissues were cultured at 37 °C, 5% CO_2_, with medium changes twice per week. The chemical structure of the compounds was shown previously [57].

### 2.3. RNA Extraction for Sequencing and Gene Expression Analysis

We extracted the total RNA with TRI reagent (Molecular Research Center, Cincinnati, OH, USA). Briefly, 1 mL of TRI reagent was added to the pooled replicates of 3 pellets from 4 independent donors. After homogenization by a Tissue Lyser (Qiagen, Hilden, Germany) for 3 min and 5 Hz, the phase separation by 1-bromo-3-chloropropane (Sigma) in a volume ratio of 1:10 with the TRI reagent was performed. The quantity and quality of the RNA samples were measured by using Nanodrop (Thermo Scientific, Waltham, MA, USA) and Bioanalyzer (Agilent, Santa Clara, CA, USA). RNA sequencing experiments and further data analysis were conducted at QIAGEN Genomic Services (Hilden, Germany).

### 2.4. Library Preparation for Next-Generation Sequencing and Ingenuity Pathway Analysis

The library preparation and Next-Generation Sequencing (NGS) was performed through QIAGEN Genomic Services (Hilden, Germany). Microarray data were selectively analyzed for VA and Epi C using QIAGEN’s Ingenuity^®^ Pathway Analysis software (IPA^®^, QIAGEN Redwood City). The significant differentially expressed genes were analyzed in different categories including IPA’s downstream effect analysis, IPA’s upstream effect analysis, and the canonical pathways.

### 2.5. Gene Expression Analysis

For the reverse transcription, SuperScript VILO cDNA Synthesis Kit (Life Technologies, Carlsbad, CA, USA) was used. For confirmation of the sequencing results, quantitative real-time PCR was accomplished using the Quant Studio - 6 instrument (Life Technologies). The gene expression assays for C-X-C motif chemokine 12 (*CXCL12*), C-C motif chemokine ligand 11 (*CCL11*), interleukin 23 subunit alpha (*IL23A*), matrix metalloproteinase 12 (*MMP12*), ADAM metallopeptidase with thrombospondin type 1 motif 16 (*ADAMTS16*), interleukin 6 (*IL-6*), growth differentiation factor 5 (*GDF-5*), cartilage oligomeric matrix protein (*COMP*), cellular communication factor 2 (*CCN2*), and *18S rRNA* are listed in Table 1 (all from ThermoFisher Scientific, Waltham, MA, USA). The relative gene expression was calculated using the 2^−ΔΔCT^ quantitative method [59], with 18S ribosomal RNA as endogenous control.

RT^2^ Profiler PCR Array (Qiagen) in 96-well plates for 84 NF-κB pathway genes and 5 housekeeping genes was performed. The data of the treatment group with VA were normalized to Ctr vehicle group. The relative gene expression was calculated using the 2^−ΔΔCT^ quantitative method and normalized with the average of the 4 housekeeping genes beta actin (*ACTB*), beta 2 microglobulin (*B2M*), glyceraldehyde 3-phosphate dehydrogenase (*GAPDH*), and ribosomal protein lateral stalk subunit P0 (*RLPLP0*) as endogenous control.

### 2.6. Matrix Metalloproteinase (MMP) Activity in Supernatants of Treated and Control Vehicle Samples

Using MMP Activity Assay Kit (Abcam, Cambridge, UK), the general activity of MMP enzymes was measured in cell culture supernatants from phase III. In this technique, a fluorescence resonance energy transfer (FRET) peptide as a generic MMP activity indicator is used. After cleavage into two separate fragments by MMPs, the fluorescence is retrieved. Briefly, 25 µL of the samples were mixed with an equal volume of 2 mM 4-aminophenylmercuric acetate (APMA) working solution and incubated for 3 h at 37 °C to activate the MMPs. Then, 50 μL of MMP Green Substrate working solution were added to the sample and control wells of the assay plate. The signal was detected by a fluorescence microplate reader (Victor 3, PerkinElmer, Waltham, MA, US) at Ex/Em = 490/525 nm.

### 2.7. Immunoassay for Pro-Inflammatory Cytokine Quantification

Using the Proinflammatory Panel 1 (human) kit (MesoScale Discovery, Rockville, MD, USA), 9 cytokines that are important in inflammation response, immune system regulation, as well as other biological processes were measured. In this regard, the supernatants were collected on day 3 of the inflammatory phase III. Concentrations of secreted cytokines in supernatants, including interferon gamma (IFN-γ), IL-1β, IL-2, IL-4, IL-12, IL-13, IL-6, IL-8, and TNF-α were quantified.

### 2.8. Nuclear and Cytoplasmic Protein Extraction of Human OA Chondrocytes

To verify the effect of VA (as anti-inflammatory compound) on IL-1β/TNF-α-dependent NF-κB pathway activation, levels of key proteins in the NF-κB pathway were assessed by Western blot analysis. For this respect, after 2D expansion of *human* OA chondrocytes in tissue culture flasks (TPP, Trasadingen, Switzerland) to 80% confluency, inflammation was induced using 1 ng/mL IL-1β/TNF-α and the cells were either treated with 1 µM VA simultaneously for 15, 30, and 60 min or in the control group with the same concentration of DMSO as control vehicle. Furthermore, a control group without induction of inflammation and treated with DMSO (control positive group) was included. After the treatment, the cells were trypsinized and washed twice in 1 mL ice-cold phosphate-buffered saline (PBS). Then, 10 × 10^6^ cells were transferred to 1.5 mL microcentrifuge tubes, and the cytoplasmic and nuclear cell extracts were prepared based on the manufacture protocol (NE-PER™ Nuclear and Cytoplasmic Extraction Kit, ThermoFisher Scientific, Waltham, MA, USA). Shortly, the cell pellet was resuspended in 200 μL of ice-cold Cytoplasmic Extraction Reagent I (CER I), containing protease inhibitors. After vortexing the tube vigorously for 15 s, it was incubated on ice for 10 min. Then, 11 μL of CERII was added and the cell suspension was vigorously mixed for 5 s. The tube was incubated on ice for 1 min and centrifuged for 5 min at maximum speed in a microcentrifuge (16,000× *g*). The supernatant (cytoplasmic extract) was transferred to a pre-chilled tube and stored at −80 °C. For nuclear extraction, 100 μL ice-cold Nuclear Extraction Reagent (NER) were added to the pellets and incubated for 40 min on ice with continued vortexing for 15 s every 10 min. Extracts were centrifuged at 16,000× *g* for 10 min and the supernatant (nuclear extract) fraction immediately transferred to a pre-chilled tube for storage at −80 °C.

### 2.9. Western Blotting

The protein concentration of nuclear and cytoplasmic extracts was measured using the bicinchoninic acid assay system (ThermoFisher Scientific) with bovine serum albumin as a standard. Cytoplasmic and nuclear proteins at final amounts of 20 µg and 10 µg, respectively, per lane, were loaded and separated by polyacrylamide gel electrophoresis (7.5% gels, BioRad, Hercules, CA, USA). The separated proteins were transferred onto nitrocellulose membranes (BioRad) and incubated in blocking buffer (1X Tris Buffered Saline/Tween (TBST) with 5% *w*/*v* nonfat dry milk) for 1 h. The membranes were incubated overnight at 4 °C with primary antibodies against Inhibitor of Nuclear Factor Kappa-B Kinase Subunit alpha (IKKα), Inhibitor of Nuclear Factor Kappa-B Kinase Subunit beta (IKKβ), NF-κB (p65), Phospho-NF-κB p65 (Ser536, P-NF-κB), Nuclear factor of kappa light polypeptide gene enhancer in B-cells inhibitor alpha (IκBα), Phospho-IκBα (Ser32, P-IκBα) (all from Cell Signaling) in 1:1000 dilutions, and GAPDH (OriGene) in 1:10000 dilution for cytoplasmic protein extracts, and NF-κB (p65), P-NF-κB (P-P65), Histon 3 (H3) (Cell Signaling) for nuclear protein extracts. Membranes were washed three times with 1% PBS/Tween and were incubated with secondary antibodies including anti-rabbit IgG, Horse Radish Peroxidase (HRP)-linked antibody or anti-mouse, HRP-linked antibody (Cell Signaling Technology, Danvers, MA, USA) in 1:2500 final concentration in 5 mL blocking buffer with gentle agitation for 1 h at room temperature. After 4 times washing with TBST, the blots were prepared for chemiluminescent detection with 1× SignalFire™ ECL Reagent (ThermoFisher Scientific, Waltham, MA, USA) as HRP chemiluminescent substrate. The quantification of the chemiluminescent signal was carried out with the use of ImageJ software [60].

### 2.10. Statistical Analysis

Statistical analysis for the gene expression, immunoassay, and MMP activity assays were performed using Graphpad Prism 8 (GraphPad Software, San Diego, CA, USA). One-way analysis of variance (ANOVA) followed by Dunnett’s post hoc test (multiple comparison) was utilized as non-parametric test of four independent experiments with three replicates of human chondrocytes microtissues. Differences were considered statistically significant at *p* < 0.05. All graphs are shown as box plots.

For the RNA sequencing dataset, the analysis was based on *q*-values. *q*-values are *p*-values that have been adjusted using the Benjamini-Hochberg False Discovery Rate (FDR) approach to correct for multiple testing. Fold changes with *q*-values below 0.05 were considered significant (*q* < 0.05).

IPA statistical analysis was based on two metrics: *p*-value and z-score. The *p*-values were provided by Fisher’s exact test (right tailed) (*p*-value ≤ 0.05 was considered significant association). The biological functions that were expected to be increased or decreased according to the gene expression changes in our dataset were identified using the IPA regulation z-score algorithm. The level of the calculated z-score reflects the overall predicted activation state of the regulator (<0: inhibited, >0: activated). A positive or negative z-score value indicates that a function is predicted to be increased or decreased in VA and Epi C treatment groups relative to control vehicle group (values above 2 or below −2 were considered as significant).

For the RT^2^ Profiler PCR Array for the NF-κB signaling pathway, the *p*-values were calculated based on a Student’s *t*-test of the replicate 2^−ΔCT^ values for each gene in the treatment groups versus the control group (*p* < 0.05).

For the Western blot bands quantification, two-way analysis of variance (ANOVA) followed by Tukey’s post hoc test (multiple comparison) was used as non-parametric test of three independent experiments with different human osteoarthritic chondrocytes.

## 3. Results

### 3.1. Whole-Genome RNA Sequencing and Gene Expression for the Differentially Expressed Genes

After screening of 34 small molecules extracted from XLGB capsule in different concentrations [57], the six most potent compounds including VA (1 µM), 5-HMF (25 µM), 4-HBA (25 µM), PS (1 µM), PCA (1 µM), and Epi C (25 µM) were selected for Next-Generation Sequencing (NGS). To identify the effect of the drugs versus untreated control group, osteoarthritic chondrocytes from four independent human donors were tested for whole-genome sequencing. Analysis of the sequencing results showed that about 47 genes were differentially expressed in each treatment group compared with the control group (Table S1).

#### Gene Expression Analysis

**Figure 1:**
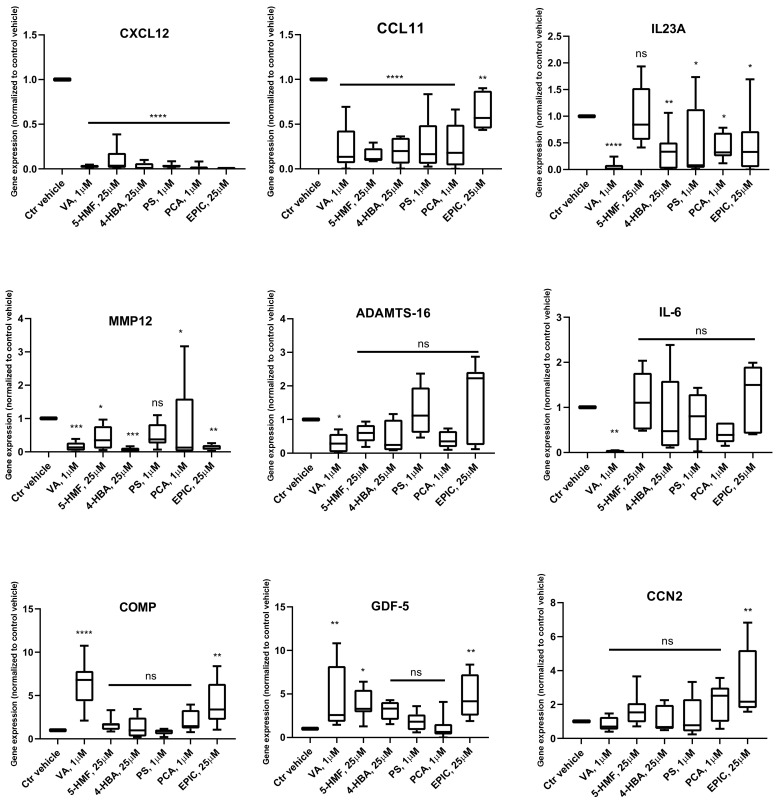
qPCR data showing transcriptional levels of genes differentially expressed in RNA sequencing dataset in compound treated versus control vehicle (Ctr vehicle) group in *human* osteoarthritis (OA) chondrocytes. *n* = 4; *n* indicates the number of *human* OA donors; for each donor, three experimental replicates were analyzed. Data are normalized to the levels of control groups. For statistical analysis using Graphpad Prism, one-way analysis of variance (ANOVA) followed by Dunnett’s *post hoc* test (multiple comparisons) was applied. * *p* < 0.01, ** *p* < 0.001, *** *p* < 0.0005, **** *p* < 0.0001 vs. Ctr vehicle, ns (non-significant).

For the confirmation and validation of RNA sequencing data, real-time RT-PCR analysis was performed for selected genes, including *CXCL12*, *CCL11*, *IL23A*, *MMP12*, *ADAMTS16*, *IL-6*, *COMP*, *GDF-5*, and *CCN2* (Figure 1). The data showed that after treatment with all the six compounds, the expression of *CXCL12* and *CCL11* was significantly inhibited in comparison with the Ctr vehicle group. Moreover, for *IL23A* and *MMP12*, all the compounds, except 5-HMF and PS, could significantly reduce the gene expression, respectively. In the treatment group with VA, the gene expression of *ADAMTS16* and *IL-6* was significantly inhibited, while the expression of *COMP* and *GDF-5* was significantly increased. Furthermore, after treatment with Epi C, the expression of *COMP*, *GDF-5*, and *CCN2* was significantly upregulated.

### 3.2. Production of Cytokines in the Groups Treated with the Small Molecules Versus Control

**Figure 2:**
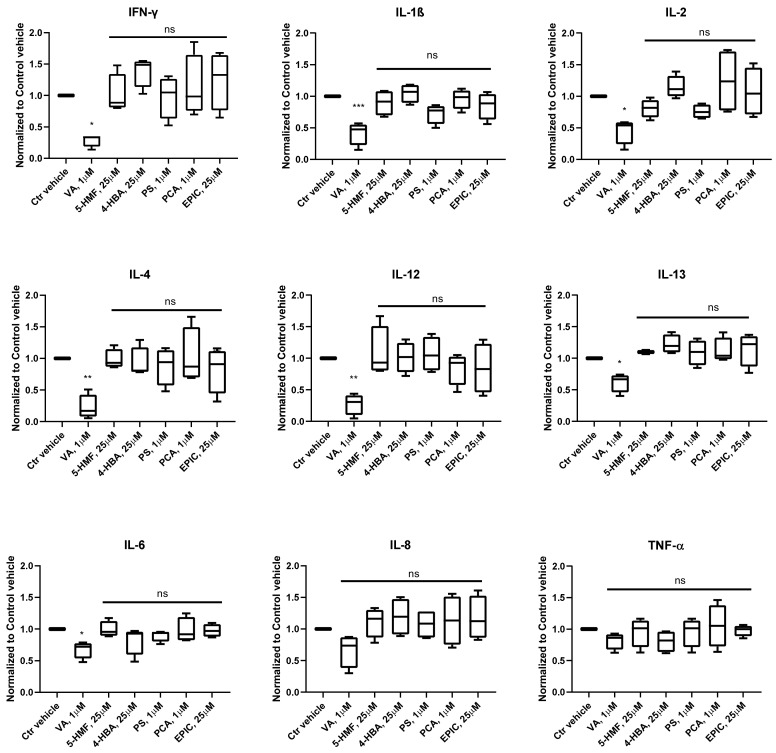
Expression profile of pro-inflammatory cytokines after treatment with Vanillic acid (VA), 5-Hydroxymethylfurfural (5-HMF), 4-Hydroxybenzoic acid (4-HBA), Psoralidin (PS), Protocatechuicaldehyde (PCA), and Epimedin C (Epi C) versus control vehicle (Ctr vehicle) group in the conditioned medium of osteoarthritic chondrocytes microtissues. The cytokines interferon gamma (INFγ), interleukin 1β (IL-1β), IL-2, IL-4, IL-12, IL-13, IL-6, IL-8, and tumor necrosis factor α (TNF-α) in phase III were measured using multiplex immunoassay. *n* = 4; *n* indicates the number of *human* OA chondrocytes donors; for each donor, three experimental replicates were analyzed. For the statistical analysis using Graphpad Prism, one-way analysis of variance (ANOVA) followed by Dunnett’s *post hoc* test (multiple comparisons) was applied. * *p* < 0.01, ** *p* < 0.001, *** *p* < 0.0005 vs. Ctr vehicle.

The concentration of inflammatory cytokines was measured in the conditioned medium of OA chondrocytes microtissues in phase III, which were treated with VA, 5-HMF, 4-HBA, PS, PCA, Epi C and normalized to control vehicle group. The results showed that in the treatment groups with VA, the cytokines including INFγ, IL-1β, IL-2, IL-4, IL-12, IL-13, IL-6, IL-8, and TNF-α were inhibited, and for all of them except for IL-8 and TNF-α the inhibition was significant (Figure 2).

### 3.3. MMP Activity of the Groups Treated with the Small Molecules Versus Control Vehicle

**Figure 3:**
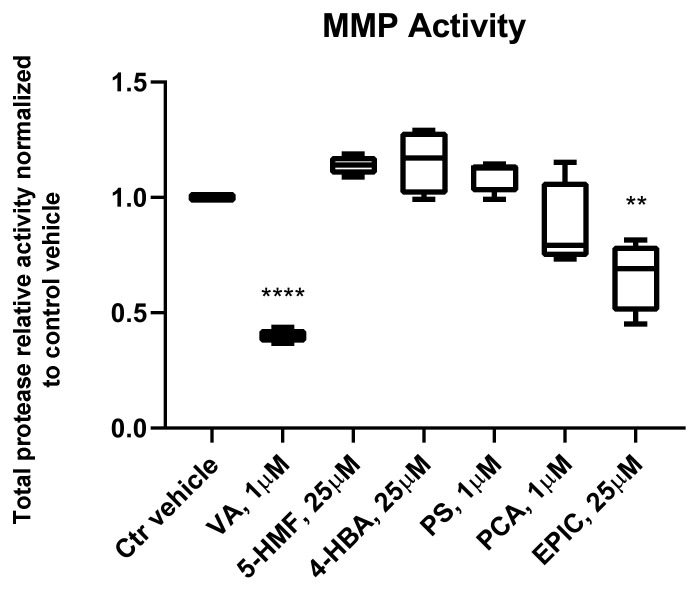
Matrix metalloproteinase (MMP) activity assay for the treated groups with VA, 5-HMF, 4-HBA, PS, PCA, and Epi C versus control vehicle (Ctr vehicle) group. MMP activity in the conditioned medium of osteoarthritic chondrocytes microtissues was measured in phase III. For the treatment groups with VA and Epi C MMP activity was significantly decreased compared to Ctr vehicle group. Total protease activity as the average relative fluorescence units (RFU) of 4 independent donors is shown. *n* = 4; *n* indicates the number of *human* OA chondrocytes donors; for each donor, three experimental replicates were analyzed. For statistical analysis using Graphpad Prism, one-way analysis of variance (ANOVA) followed by Dunnett’s *post hoc* test (multiple comparisons) was applied. ** *p* < 0.001, **** *p* < 0.0001 vs. Ctr vehicle.

MMPs are the key enzymes for the breakdown of connective tissues and play an important role in the development of OA. MMP activity for the groups treated with VA, 5-HMF, 4-HBA, PS, PCA, and Epi C versus control vehicle group was measured in the conditioned medium of osteoarthritic chondrocytes microtissues in phase III. Total protease activity for the treatment groups with VA and Epi C was significantly decreased compared to the control vehicle group (Figure 3).

### 3.4. Ingenuity Pathways Analysis (IPA)

Among all the treatment groups, VA and Epi C showed the most significantly differentially expressed genes and proteins in all tested donors. Therefore, data of whole-genome sequencing of these two compounds versus their control negative groups were selected for Ingenuity Pathway Analysis (IPA). In general, the results of the Core analysis of the VA and Epi C data sets were driven by a remarkable downregulation of different cytokines and chemokines as well as a substantial upregulation of ribosomal genes and translation elongation/initiation factors.

#### 3.4.1. IPA’s Downstream Effect Analysis

**Figure 4:**
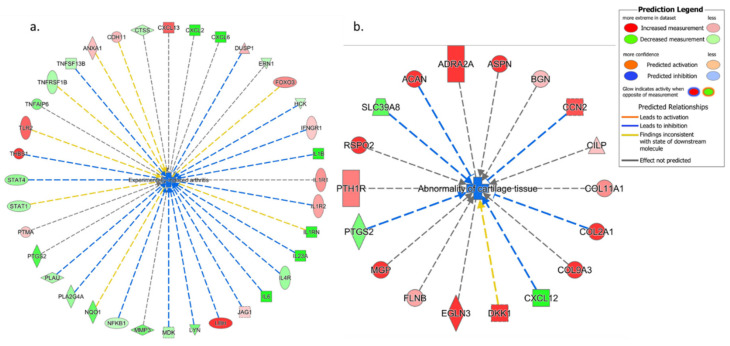
Ingenuity Pathways Analysis (IPA) showing top connective tissue disorders from the table of low-level (specific) process or functions. (**a**) Example network of the term “Experimentally induced arthritis” for VA vs. Ctr group, which was composed of mostly downregulated cytokines, chemokines, and nuclear factor kappa-light-chain-enhancer of activated B cells (NF-κB) downregulation. (**b**) Example network of the term “Abnormality of cartilage tissue” for Epi C vs. Ctr group, which showed upregulation of collagens, biglycan, CCN2, and SOX9. Red represents upregulation of genes, while green represents downregulation of genes. Light red or green represent slight upregulation or downregulation.

The results of IPA’s downstream effect analysis for the high-level categories of biological processes demonstrated several relevant categories including Inflammatory Disease, Inflammatory Response, Immune Cell Trafficking, Connective Tissue Disorder, Protein Synthesis, and Cellular Growth/Proliferation on top of the list for both VA and Epi C datasets (Figure S1a,b). In the low-level (specific) processes or functions that belong to the high-level category “Connective Tissue Disorder” for the VA vs. control (Ctr) group, 4 terms with significant negative activation z-scores, including inflammation of the joint, rheumatic disease, experimentally induced arthritis, and chondrodysplasia, indicated a significant decrease of all these functions. For the Epi C vs. Ctr group, inflammation of the joint and abnormality of cartilage tissue were top results with negative z-scores (predicted decrease, non-significant) (Table S2a,b). From the table of top connective tissue disorders (Table S2a), the network of the term “Experimentally-induced arthritis” for VA vs. Ctr group was composed of mostly downregulated cytokines and chemokines. Some differentially expressed components of ECM, collagens, MMPs, ADAMTSs, CCN2 upregulation, and NF-κB downregulation were also observed (Figure 4a). Moreover, from the table of top connective tissue disorders, the network of the term “Abnormality of cartilage tissue” for Epi C vs. Ctr group showed upregulation of collagens, biglycan (BGN), CCN2, and SRY-Box Transcription Factor 9 (SOX9) (Figure 4b). Furthermore, for VA vs. Ctr group, the table of low-level (specific) process or functions that belong to the high-level category “Inflammatory Disease” showed similar results to “Connective Tissue Disorder”. Finally, most of the terms for the table of “Immune Cell Trafficking” showed significantly negative activation z-scores that strongly indicated a reduced activation, migration, chemotaxis, or movement of different immune cell types. Example network of the term “Activation of Lymphocytes” as the top disorder of “Immune Cell Trafficking” was dominated by downregulated cytokines, chemokines, and downregulated NF-κB1 (Figure S2a,b). For Epi C vs. Ctr treatment group, the table of low-level (specific) process or functions that belong to the high-level category “Inflammatory Disease” showed negative activation z-scores for the terms Inflammation of the Joints, Rheumatic Disease and Chronic Inflammatory Disorder, which suggested a moderate decrease of these functions or processes. The example of network for the term “Inflammation of the Joints” as a decreased function was supported by the downregulated chemokines/cytokines as well as upregulated ECM components such as COL2A1, BGN, and CCN2 (Figure S3a,b). On the other side, IPA also predicted a mild increase of protein translation and synthesis, which might point to an increase of anabolic activities in the microtissues treated with VA and Epi C. In this regard, several components of the ECM were found to be upregulated, such as collagen type V alpha 1 (COL5A1), collagen type VI alpha 3 (COL6A3), collagen type VIII alpha 1 (COL8A1), biglycan (BGN), laminin alpha 2 (LAMA2), laminin alpha 3 (LAMA3), laminin beta 1 (LAMB1), and laminin gamma 1 (LAMC1) (Figure S4a,b). Furthermore, IPA upstream regulators were filtered for the proteins that could explain the upregulation of ECM components in VA and Epi C treatment groups vs. Ctr vehicle. The data revealed that CCN2 was activated, which could explain the upregulation of ribosomal genes and translation initiation/elongation factors. The example of the upstream regulator network of CCN2 showed the upregulation of many ECM components (Figure S5a,b).

#### 3.4.2. IPA’s Upstream Regulator Analysis

**Figure 5:**
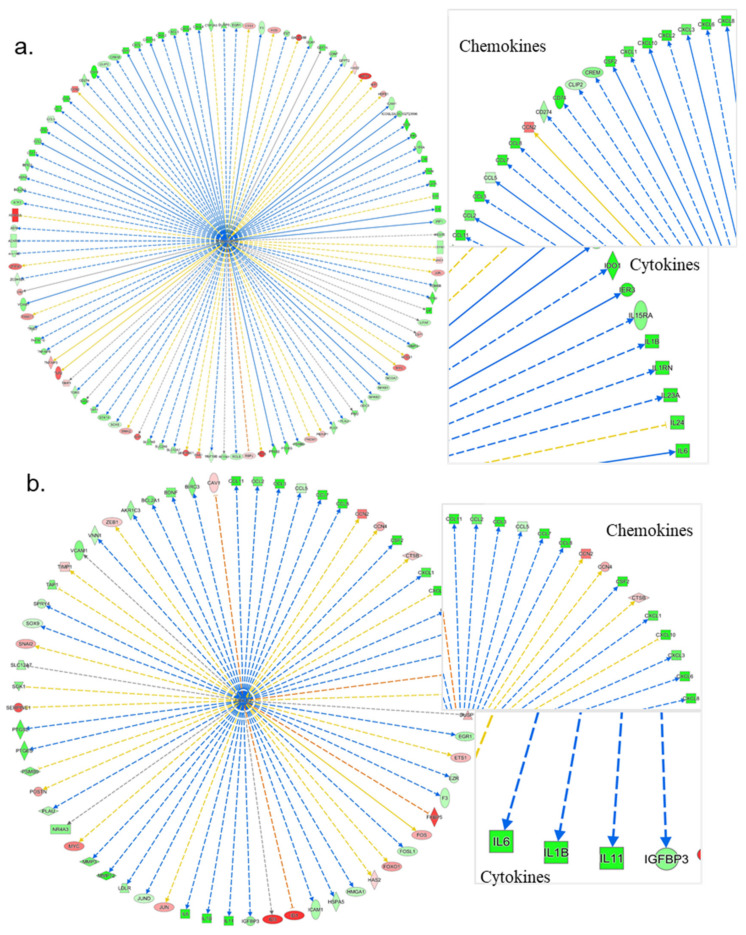
Ingenuity Pathways Analysis (IPA) showing upstream regulators for NF-κB and ERK1/2 for VA vs. Ctr treatment group predicted to be inhibited. The predicted inhibition of these putative upstream regulators largely agrees with the observed downregulation of numerous chemokines and cytokines. (**a**) Upstream regulator network for an inhibited NF-κB complex. (**b**) Upstream regulator network for an inhibited ERK1/2 complex.

Gene expression changes in the chondrocytes treated with VA and Epi C showed an anti-inflammatory response. This was supported not only by the observed downregulation of various cytokines but also by IPA’s upstream regulator analysis, which suggested that parts of the observed gene expression changes were strongly reminiscent of effects of different anti-inflammatory or immunosuppressive compounds on transcriptomes. More specifically, in the treatment group with VA, different small molecule compounds, known to interfere with the activity of MEK/MAP or PI3 kinases, were found in the list of putative upstream regulators (Table S3). In addition, the predicted downregulation of NF-κB or extracellular-signal-regulated kinase (ERK1/2) (Figure 5a,b) and upregulation of IL-10 as anti-inflammatory cytokine, could account for the observed reduction of cytokine/chemokine expression as well as the anti-inflammatory expression pattern (Figure S6).

For the treatment groups with Epi C, gene expression changes in the treated chondrocytes also suggested an anti-inflammatory response. This was not only supported by the observed downregulation of various cytokines but also by IPA’s upstream regulator analysis, showing that the observed gene expression changes were strongly reminiscent of effects of an anti-inflammatory compound, dexamethasone, on transcriptomes (Table S4). Furthermore, the upstream regulator table showed that TNF, IFN-γ, and Signal transducer and activator of transcription 1 (STAT1) were the top inhibited upstream regulators, and the upstream regulator network for the inhibited STAT1 showed several chemokines and cytokines were downregulated (Figure S7).

#### 3.4.3. The Canonical Pathways

**Figure 6:**
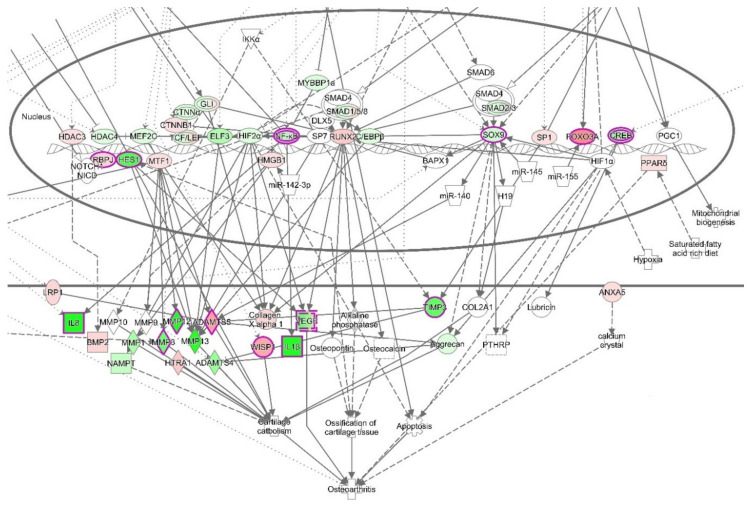
The canonical pathway “Osteoarthritis Signaling” for VA vs. Ctr treatment group. In this instance, the comparison of expected node activities and actual measurements suggest a reduced activity of this canonical pathway (*p*-value: 5.5 × 10^−12^ z-score: −0.707). Noteworthy is the downregulation of NF-κB (expected activation) and inflammatory markers including IL-1β. Red fillings = “activated”, green fillings = “inhibited”, purple highlights = passed cut-offs, no highlight = did not pass cut-offs.

**Figure 7:**
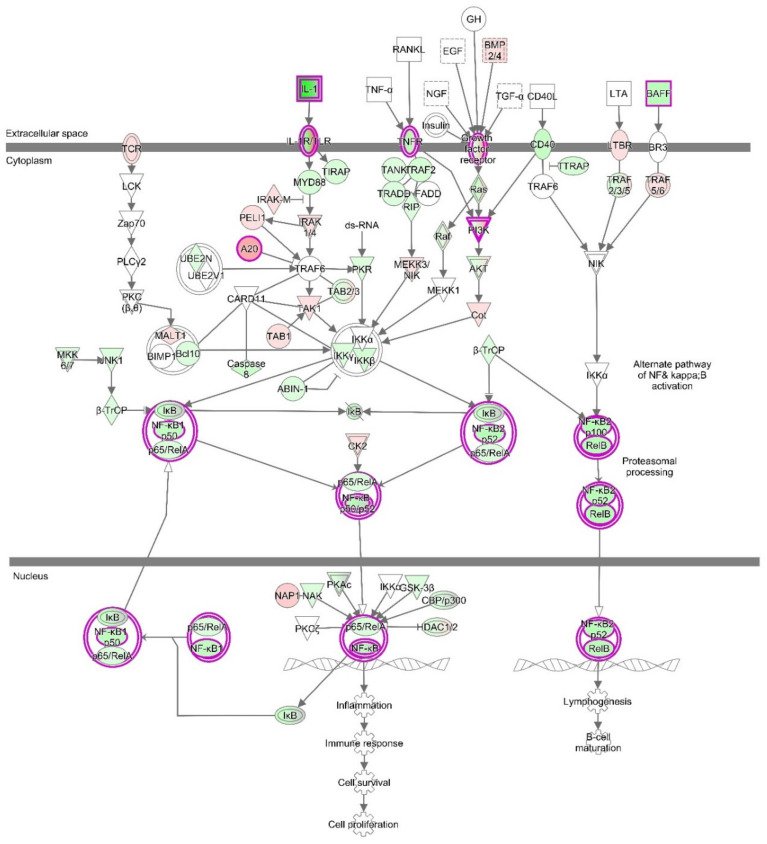
The canonical pathway “NF-kB Signaling” for VA vs. Ctr treatment group. This canonical pathway has a negative activation z-score (*p*-value = 4.25 × 10^−4^, z-score = −0.5, tendency for inhibition). Red fillings = “activated”, green fillings = “inhibited”, purple highlights = passed cut-offs, no highlight = did not pass cut-offs.

Moreover, canonical pathways that include NF-κB, such as Osteoarthritis Signaling (this canonical pathway set has been described as a critical regulatory element for the onset of osteoarthritis) (Figure 6 and Figure S8), HMGB1 signaling, or NF-κB signaling (Figure S9), were predicted to be inhibited. Osteoarthritis Signaling comprises several pathways including NF-κB, P38/MAPK, ERK/MAPK, Wnt/β-Catenin, and protein kinase A signaling. By the obvious central role of a downregulated NF-κB complex and negative z-score for the NF-κB pathway, it becomes apparent that this pathway was reduced in its activity after treatment with VA (Figure 7).

**Figure 8:**
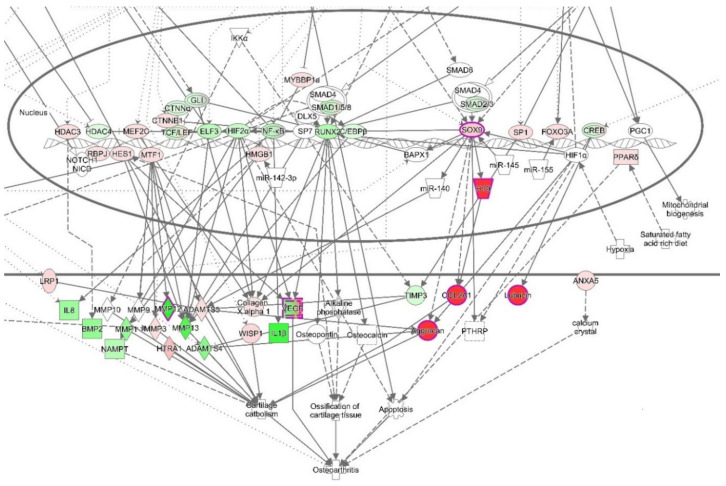
The canonical pathway “Osteoarthritis Signaling” for Epi C vs. Ctr treatment group. This canonical pathway has been described as a critical regulatory element for the onset of osteoarthritis. Based on the activation z-score (*p*-value = 1.45 × 10^−3^, z-score = −1.213), the normal function of this pathway is reduced. Noteworthy is the downregulation of NF-κB (expected activation) and the upregulation of SOX9 (expected inhibition). Red fillings = “activated”, green fillings = “inhibited”, purple highlights = passed cut-offs, no highlight = did not pass cut-offs.

For the treatment group with Epi C, Osteoarthritis Signaling was significantly affected, and the inhibition of this canonical pathway was more pronounced than in the VA treatment group vs. Ctr dataset (Figure 8 and Figure S10). In this instance, the comparison of expected node activities and actual measurements suggested a reduced activity of this canonical pathway. Interestingly, SOX9, COL2A1, and ACAN, which are key markers of cartilaginous matrix production, were upregulated while NF-κB was downregulated (Figure 8). Additionally, among the canonical pathways that overlap with molecules associated with downstream effect term “protein translation”, mTOR signaling was found to be activated. In the canonical pathway “mTOR Signaling”, the translational/ribosomal genes were upregulated, and among them, the upregulation of protein kinase C (PKC) regulated in development and DNA damage responses 1 (REDD1) were significant (Figure S11). Hence, these activations could indicate the increase in anabolic activities in Epi C vs. Ctr group.

### 3.5. RT^2^ Profiler PCR Array for the NF-κB Signaling Pathway

**Figure 9:**
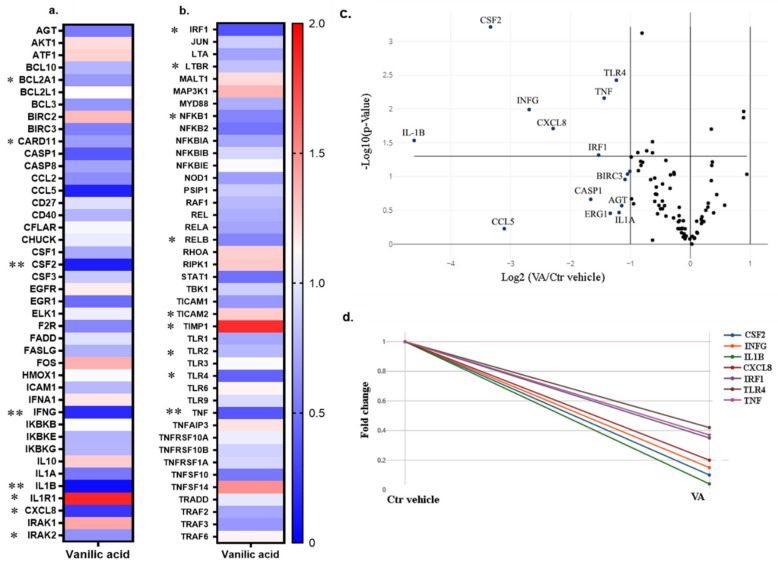
RT^2^ Profiler PCR Array for the NF-κB signaling pathway. The data of the treatment group with VA is normalized to the Ctr vehicle group. (**a**,**b**) The average of the fold change for 3 donors of *human* OA chondrocytes are shown in heat map graphs. The statistically significant genes are highlighted by the asterisk. The *p*-values are calculated based on a Student’s *t*-test of the replicate 2^−ΔCT^ values for each gene in the control group versus treatment group. * *p* < 0.05 ** *p* < 0.005. (**c**) Volcano plot for VA treatment group versus Ctr vehicle showing significantly down-regulated genes which passed −1 threshold for log2 fold change difference. (**d**) Multigroup Plot for the genes with significant *p*-values and log2 fold regulations <−1. These genes include colony-stimulating factor 2 (*CSF2*), interferon gamma (*INF*-*γ*), interleukin 1-beta (*IL1B*), interleukin 8 (*CXCL8*), Toll-like receptor 4 (*TLR4*), and tumor necrosis factor (*TNF*).

The results of the PCR array for the NF-κB signaling pathway for VA vs. Ctr vehicle group showed that many of the genes in the pathway were inhibited after treatment with VA. The data of the treatment group with VA was normalized to the Ctr vehicle group and the fold change of the VA treatment group vs. Ctr vehicle showed significantly differentially expressed genes that are marked by asterisks in the heat map graphs (Figure 9a,b). Most of the genes with significant *p*-values were downregulated. These genes included BCL2-related protein A1 (*BCL2A1*), caspase recruitment domain family 11 (*CARD11*), colony-stimulating factor 2 (*CSF2*), *INF*-*γ*, interleukin 1-beta (*IL1β*), interleukin 8 (*CXCL8*), interleukin 1 receptor-associated kinase 2 (*IRAK2*), interferon regulatory factor 1 (*IRF1*), lymphotoxin beta receptor superfamily member 3 (*LTBR*), nuclear factor of kappa light polypeptide gene enhancer in B cells inhibitor, alpha (*NFKBIA*), nuclear factor of kappa light polypeptide gene enhancer in B cells 1 (*NF-κB1*), transcription factor RelB (*RELB*), Toll-like receptor 2 (*TLR2*), Toll-like receptor 4 (*TLR4*), and tumor necrosis factor (*TNF*). Some other genes, including interleukin 1 receptor 1 (*IL1R1*), Toll-like receptor adaptor molecule 2 (*TICAM2*), and tissue inhibitor of metalloproteinase 1 (*TIMP1*) were significantly upregulated. Furthermore, the volcano plot for VA treatment group vs. Ctr vehicle showed significantly downregulated genes that passed the -1 threshold for the (log2) fold change difference (Figure 9c). The genes with significant fold-change values (*p* < 0.05) and fold-regulation values less than -2 are indicated in the Multigroup Plot (Figure 9d). These genes included *CSF2*, *INF*-*γ*, *IL1B*, *CXCL8*, *TLR4*, and *TNF*.

### 3.6. Western Blot Analysis for the NF-κB Signaling Pathway

**Figure 10:**
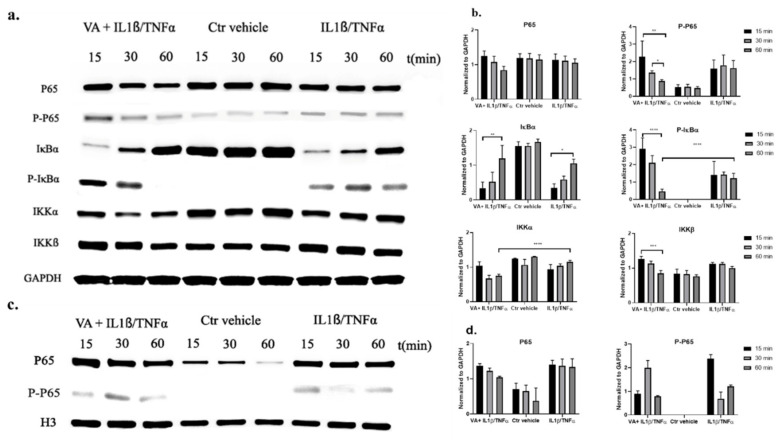
The expression of the main proteins in the NF-κB signaling pathway including IκB kinase (IKKα), IKKβ, NF-κB (P65), P-NF-κB (P-P65), IκBα, P-IκBα in treatment groups with VA versus untreated control groups was determined by Western blot analysis. Glyceraldehyde 3-phosphate dehydrogenase (GAPDH) was used as endogenous control. After induction of inflammation with 1 ng/mL IL-1β/TNF-α in *human* OA chondrocytes, the cells were either simultaneously treated with 1 µM VA for 15, 30, and 60 min or were treated with 0.01% dimethyl sulfoxide (DMSO) as the control vehicle. Furthermore, the Ctr vehicle group without induction of inflammation and treated with 0.01% DMSO (control positive group; Ctr vehicle) was probed. (**a**) The protein level of cytoplasmic P65, P-P65, IκBα, P-IκBα, IKKα, IKKβ determined by Western blot with GAPDH as endogenous control. (**b**) Quantification of the protein band intensity. The intensity of each protein was normalized to GAPDH. (**c**) The protein level of nuclear P-65 and P-P65 determined by Western blot with histone 3 (H3) as endogenous control. (**d**) Quantification of nuclear P65 and P-P65 protein band intensity. The intensity of each protein was normalized to H3. For statistical analysis using GraphPad Prism, two-way analysis of variance (ANOVA) followed by Tukey’s *post hoc* test (multiple comparisons) was applied for the treated groups with VA vs. Ctr vehicle and IL-1β/TNF-α groups in three independent experiments with different donors (*n* = 3). * *p* < 0.01, ** *p* < 0.001, *** *p* < 0.0005, **** *p* < 0.0001.

To further investigate the effect of VA on the NF-κB signaling pathway, the expression of the main proteins in the pathway, including IKKα, IKKβ, NF-κB (P65), P-NFκB (P-P65), IκBα, P-IκBα, were determined. In the cytoplasmic extracts, the expression of P-P65, P-IκBα, and IKKβ was significantly decreased after 60 min of treatment with VA + IL-1β/TNF-α compared with the 15 min treatment groups; while these three proteins remained constant over time in the IL-1β/TNF-α group. Furthermore, the expression of P-IκBα was significantly decreased after 60 min of treatment with VA compared with the inflammatory untreated control group. On the other hand, the expression of IκBα protein was increasing similarly over time in both the VA + IL-1β/TNF-α and IL-1β/TNF-α groups. After induction of inflammation, the amount of IκBα was reduced due to the phosphorylation leading to its rapid degradation. However, after 60 min, IκBα was resynthesized and this effect was reversed. In the control vehicle group, the expression of the proteins at different time points remained intact. Moreover, the reference protein (GAPDH) was unaffected in all the experimental groups (Figure 10a,b). In the nuclear extracts, the P65 protein in the treatment group with VA showed slightly decreased expression after 60 min treatment with the drug, while it was not different over time in the inflammatory untreated control group. In the untreated control group, less expression of P65 in the nucleus was observed compared with the inflammatory groups though this was not significant. The reference protein histone 3 (H3) was unaffected in all the experimental groups (Figure 10c,d).

## 4. Discussion

To have an effective treatment against OA, inhibition of pro-inflammatory cytokines that are excessively abundant in osteoarthritic joints is necessary. Furthermore, for regeneration of damaged cartilage, it is essential to increase the chondrocytes anabolism to rebuild cartilage and restore joint function. Inflammatory cytokines including IL-1β and TNF-α initiate the development and progression of OA through activating or inhibiting different signaling pathways such as NF-κB, HMGB1, IL-1R/TLR, MAPKs, and PI3K/Akt/mTOR pathways [8,9,10,11,12,13,14,15,16,17,18]. In our previous study, we used these two inflammatory cytokines on microtissues of *human* primary chondrocytes to develop an inflammatory model of arthritis. After screening of 34 bioactive compounds existing in the over-the-counter XLGB formula in an inflammatory model, we identified six compounds (VA, 5-HMF, 4-HBA, PS, PCA, Epi C) with anti-inflammatory and anabolic properties [57]. In the current study, RNA sequencing, gene expression, and immunoassay analyses further demonstrated that several pro-inflammatory cytokines, which are abundant in arthritic joints, were significantly downregulated and in the treatment groups with VA and Epi C, the genes for the ECM protein synthesis were significantly upregulated (Figure 1, Figure 2). Furthermore, the MMP activity was significantly suppressed in these treatment groups, indicating anti-catabolic effects of VA and Epi C (Figure 3). The upregulation of *TIMP1* as a strong inhibitor of MMPs in the treatment group with VA supported its significant inhibitory effect on MMPs (Figure 9). Therefore, we selected these two most potent compounds (VA, Epi C) for the pathway analysis.

IPA showed that after treatment with VA, osteoarthritic signaling including NF-κB pathway was inhibited (Figure 6 and Figure 7). NF-κB regulates the expression of genes involved in inflammatory responses, and several studies have shown that inhibition of NF-κB pathway can decrease the pathogenesis of OA and RA [25,26,61,62]. Recently, several TCM compounds including VA have shown potential in inhibiting inflammatory response through inhibition of different parts of the NF-κB pathway [48,63,64,65,66,67,68,69,70]. An in vitro study on lipopolysaccharide (LPS)-stimulated *mouse* peritoneal macrophages showed that VA had anti-inflammatory effects that were mediated by the inhibition of LPS-induced NF-κB activation and IκB-α degradation [71]. Moreover, an in vivo study on a murine model of inflammatory pain showed that VA could inhibit pro-inflammatory cytokine production by suppressing NF-κB activity. Our data suggest that after induction of inflammation in *human* OA chondrocytes and activation of NF-κB pathway, the treatment with VA affected the IKK complex, resulting in reduction of IκBα protein phosphorylation over time (Figure 10a,b). In the inactive NF-κB signaling pathway, IκBα protein is bound to the NF-κB dimers which leads to cytoplasmic retention of the NF-κB complex. Phosphorylation of IκBα protein initiated by inflammatory cytokines leads to its degradation and the NF-κB dimers would release and translocate to the nucleus, initiating the transcription of genes involved in inflammation. Yin et al. also showed that anti-inflammatory small molecules such as Aspirin^®^ or sodium salicylate specifically inhibit IKK-β activity. The mechanism of Aspirin^®^ or sodium salicylate in inhibition of IKK is due to binding of these compounds to IKK-β and competing with ATP binding [72]. VA, which is the extract of *Radix ET Rhizoma Salviae*, has a molecular structure similar to salicylates; we therefore predict that this compound may be acting by binding to the IKK-α, IKK-β and competing with ATP binding, which subsequently results in inhibition of P-IκBα.

Recent studies showed that HMGB1 could promote the pathogenesis of inflammatory diseases including arthritis [73,74,75]. HMGB1, which is secreted during necrotic cell death and activation of macrophages, is very abundant in synovitis and intra-articular fluid of RA patients. The IPA dataset for the treatment group with VA indicated that HMGB1 signaling was inhibited (Supplementary Figure S8). HMGB1 triggers the signal to the nucleus by activating several mitogen-activated protein kinases leading to nuclear translocation of NF-kB for initiating an inflammatory response. It can act either alone or in combination with other inflammatory cytokines (INF-γ, IL-1β, TNF-α), which leads to an increased inflammatory signal [76]. The IPA dataset for the VA treatment group predicted that INF-γ and TNF receptors were significantly inhibited. Therefore, inhibition of INF-γ, IL-1β, and TNF receptors could be a successful approach in inhibition of the inflammatory response. Furthermore, upstream regulator analysis for compounds and drugs revealed that VA may act in a similar way like small molecule compounds that are known to interfere with the activity of MEK and PI3 kinases (Table S3) [77,78,79]. Most of the established kinase inhibitors are competitive inhibitors of ATP and target the ATP-binding pocket [80,81,82,83,84]. In further studies, it will be important to understand the specific mechanism of action of VA as a competitive inhibitor towards the development of a drug with fewer side effects than NSAIDs.

**Figure 11:**
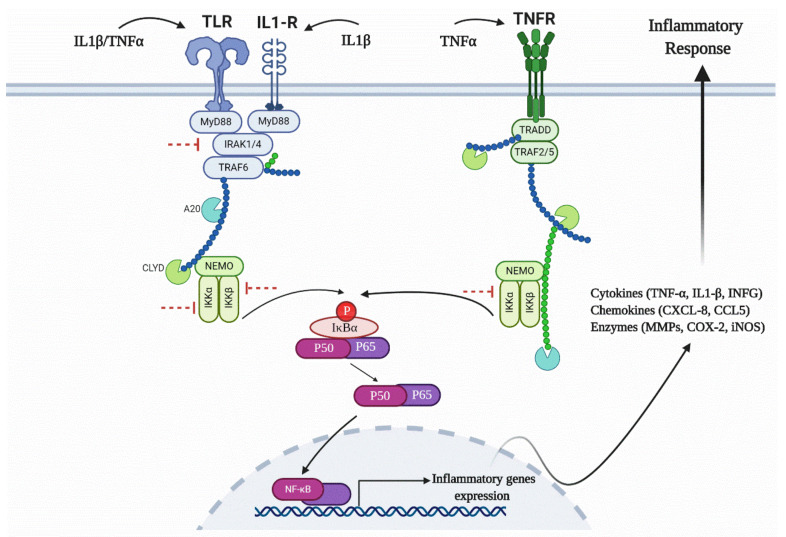
Schematic figure of the NF-κB signaling pathway and inhibition of IKK complex after treatment with VA. The red inhibitor arrows show the predicted inhibitory effect of VA in different parts of the pathway.

The Toll-like receptors (TLR)/interleukin-1 receptor (IL-1R) or TIR superfamily of receptors are also activated by IL-1β and TNF-α. These receptors including TLR1, TLR2, TLR4, TLR5, and TLR6, which are present in mono or heterodimeric structures, are important in cartilage pathologies [85,86]. TLRs are expressed in OA synovial tissues and various endogenous ligands are present within the inflamed joints of OA patients [10,87]. HMGB1 is one of the most known endogenous TLR4 ligands involved in OA pathology. After interaction of the ligands with their receptors, they connect with one or more adaptor proteins. These adaptors, including Myeloid differentiation factor 88 (MyD88), the TIR domain-containing adaptor protein inducing interferon-β (IFNβ) (TRIF; also known as TICAM1), and the TRIF-related adaptor molecule (TRAM; also known as TICAM2) are connected to the cytoplasmic domains of the receptors. MyD88 recruits the members of the interleukin-1 receptor-associated kinase (IRAK) family (IRAK1, IRAK2, and IRAK4) [88,89,90,91]. Phosphorylated IRAK2 possesses kinase activity that reacts with TNF-receptor-associated factor 6 (TRAF6) and recruits transforming growth factor β-activated kinase 1 (TAK1). Activated TAK1 stimulates the IKK complex, which initiates phosphorylation, and following degradation of IκBα, leads to the activation of NF-κB for translocation from the cytosol to the nucleus and activation of an inflammatory response. Our PCR array dataset showed that several genes associated with this pathway including *IRAK2*, *TLR2*, and *TLR4* were significantly downregulated. Furthermore, TNF regulates the expression of the transcription factor interferon regulatory factor 1 (*IRF1*) [92], which was significantly inhibited after treatment with VA (Figure 9). Therefore, the TIR superfamily of receptors that contribute to IKK phosphorylation and NF-κB activation may be a target for VA and its anti-inflammatory properties (Figure 11).

All the mentioned signaling pathways are interconnected and act through inhibition of the canonical NF-κB pathway, which is activated by inflammatory signals, while the non-canonical NF-κB pathway, which is activated by developmental signals and is necessary for developmental gene expression, is still active [93]. Therefore, after treatment with VA, the expression of inflammatory cytokines including IL1-β, TNF, and INF-γ were significantly inhibited. Interestingly, the *IL1R1* was significantly upregulated in the treatment group compared with the inflammatory control group. However, previous studies also showed an inverse relation between *IL1R1* expression and the amount of IL-1β in OA joints. Wyatt et al. showed that *IL1R1* was downregulated in OA synovium compared to non-arthritic controls [94]. Moreover, in retinal endothelial cells, *IL1R1* expression was downregulated during activation by IL-1β [95]. Furthermore, clinical trials with OA patients using IL1 receptor antagonist did not show symptomatic improvements compared with placebo, which could be due to the downregulation of *IL1R1* prior to the treatment [96,97].

Furthermore, IL-1β and TNF-α suppress the expression of genes related to the differentiated chondrocyte phenotype, including *COL2A1* and *ACAN* [2,5]. In our previous study, we showed that after treatment with Epi C, not only catabolic marker genes (*MMPs*) were significantly inhibited but also anabolic marker genes (*COL2A1* and *ACAN*) were significantly upregulated [57]. RNA sequencing and gene expression data revealed that besides *COL2A1* and *ACAN* also other anabolic genes including *GDF-5*, *COMP*, and *CCN2* were significantly upregulated in the treatment group with Epi C (Figure 1). GDF-5 is an important growth factor acting as an extracellular signaling molecule in the formation and repair of joints [98]. COMP is a crucial structural and functional component of the cartilage ECM, which interacts with other ECM proteins and facilitates the interaction of chondrocytes with the ECM [99]. CCN2 plays an important role in promoting growth and differentiation of auricular chondrocytes in vitro and in vivo. Besides, CCN2 can increase proteoglycan synthesis but does not promote hypertrophy or calcification of articular chondrocytes [100,101]. Furthermore, osteoarthritic signaling pathway and several inflammatory marker genes in the pathway were inhibited in the Epi C group (Figure 8). Therefore, Epi C showed significant anabolic and anti-catabolic effects, which play an important role in regeneration of cartilage.

There is very limited literature available on the anabolic effect of Epi C (as *herba Epimedii* extract), which has been used as TCM for the treatment of osteoporosis for many years [102]. Epi C and icariin are the major flavonoid glycosides existing in *herba Epimedii*, and several studies revealed that the flavonoids of *Epimedii* could regulate signaling pathways associated with bone and cartilage repair [52,103,104,105]. Icariin, as a well-studied small molecule with similar chemical structure as Epi C, showed great potential in cartilage repair in in vitro and in vivo studies [52,106]. An in vitro and in vivo study in murine showed that icariin may act as a hypoxia inducible factor (HIF)-1α activator, which could promote chondrocyte proliferation, differentiation, ECM synthesis, and articular cartilage repair [106]. Furthermore, the effect of icariin in inhibition of cathepsin K and attenuation of bone and cartilage degradation in a murine model of collagen-induced arthritis and its anti-inflammatory effect in an LPS induced inflammatory murine model were reported [107,108]. In our IPA dataset, mTOR signaling, which is responsible for the proliferation and protein translation, was predicted to be activated [109] (Figure S11). It was shown that PI3K/AKT/mTOR signaling was necessary for cartilage homeostasis and in human OA cartilage, this pathway was downregulated compared with normal cartilage [18,110]. Previous studies showed that some drugs could attenuate apoptosis and degradation in IL-1β-induced chondrocytes by promoting cell survival and matrix protein synthesis through activation of PI3K/AKT/mTOR signaling pathway [18,79,111,112,113]. Therefore, the predicted activation of mTOR signaling could explain the protective and anabolic effects of Epi C on chondrocytes versus Ctr inflammatory group.

Due to the complexity of the different signaling pathways and their interconnections with each other, the exact intervention of the drug with the target often remains undefined. The classical approaches including inhibition at the level of chemokines/cytokines and receptors with small molecules or antibodies are often insufficient interventions since they block one ligand or receptor, while the associated intracellular signals remain active through other present ligands or receptors. However, targeting the kinase activity is one of the most novel and effective strategies in inhibition of inflammatory responses with promising effects [72,81,83]. Nevertheless, many kinases are involved in several signaling pathways, which creates concerns regarding undesired side effects of the inhibition or activation of kinases. So, specificity of the drug in inhibition or activation of kinases in the pathways that are involved in the activation of pro-inflammatory mediators or inhibition of matrix protein synthesis is of great importance. Local drug delivery to the OA joint is another strategy to prevent systemic side effects.

An investigation on the pharmacokinetic and oral bioavailability of Epi C showed that if the drug was administered as a pure compound, its effect was about four-fold higher than that of the complex *herba Epimedii* extracts [114]. The oral bioavailability of Epi C in *herba Epimedii* extract might be affected by the other herbal ingredients suppressing Epi C absorption from the gastrointestinal system. These results highlight the importance of discovering the most potent compounds in the complex herbal extracts toward having a potent drug (or combinations of selected drugs) in which their effects are not masked by other existing compounds in the herbal mixture. Furthermore, formulation of a local drug delivery system can be more easily achieved for a single or combination of the most potent compounds.

In our study, *human* OA chondrocytes as the most clinically relevant source of cells for testing the potent compounds in OA treatments were used. Nevertheless, using this source of cells has some limitations. To have the least amount of heterogeneity in the OA cell population, *human* cells were isolated from macroscopically evident OA areas of the joint; therefore, limited numbers of primary cells were acquired, and passage three chondrocytes were used that might have affected the original OA traits. However, to minimize the cell de-differentiation an established expansion medium supplemented with TGF-β and FGF-2 was used that promotes cell proliferation and maintains cell differentiation capacity [115]. Furthermore, by using IL-1β/TNF-α as inflammatory cytokines, our microtissue model resembled post-traumatic acute inflammation which is not the exact condition in OA characterized by chronic inflammation.

## 5. Conclusions

In conclusion, for the first time, the mechanism of action of two potent herbal compounds (VA, Epi C) existing in the XLGB formula was investigated on *human* osteoarthritic chondrocytes using an ex vivo approach. VA exhibited an important role in attenuation of inflammation by affecting I-κBα phosphorylation in the NF-κB signaling pathway and Epi C showed significant anabolic effects with increasing collagenous and non-collagenous matrix proteins for regeneration of cartilage. Therefore, these drugs may attenuate cartilage degeneration through regulation of the tissue hemostasis. In further pre-clinical studies, the synergic effects of VA with anti-inflammatory properties and Epi C with anabolic activity warrants to be investigated.

## Figures and Tables

**Table 1:** Gene expression assays (*human*) used for real-time PCR.

Gene	Probe Type	Assay ID
*CXCL12*	5′ FAM-3′ NFQ	Hs03676656_mH
*CCL11*	5′ FAM-3′ NFQ	Hs00237013_m1
*IL23A*	5′ FAM-3′ NFQ	Hs00372324_m1
*MMP12*	5′ FAM-3′ NFQ	Hs00159178_m1
*ADAMTS16*	5′ FAM-3′ NFQ	Hs00373526_m1
*IL-6*	5′ FAM-3′ NFQ	Hs00174131_m1
*COMP*	5′ FAM-3′ NFQ	Hs00164359_m1
*GDF-5*	5′ FAM-3′ NFQ	Hs00167060_m1
*CCN2*	5′ FAM-3′ NFQ	Hs00170014_m1
*18s fast*	5′ FAM-3′ NFQ	Hs99999901_s1

*CXCL12*: C-X-C motif chemokine 12, *CCL11*: C-C motif chemokine ligand 11, *IL23A*: interleukin 23A, *MMP12*: matrix metalloproteinase 12, *ADAMTS16*: A Disintegrin and Metalloproteinase with Thrombospondin motifs 16, *IL-6*: interleukin 6, *GDF-5*: growth differentiation factor 5, *COMP*: cartilage oligomeric matrix protein, *CCN2*: connective tissue growth factor, and *18S rRNA* (all from ThermoFisher Scientific). FAM: Carboxyfluorescein; NFQ: nonfluorescent quencher.

## Supplementary Materials

The following are available online at https://www.mdpi.com/2218-273X/10/6/932/s1, Figure S1: Top diseases and biological functions from the core analysis. Figure S2: Low-level (specific) process or functions belonging to the high-level category “Immune cell trafficking”, Figure S3: Example network of the term “Inflammation of the joints”, Figure S4: Example network of the term “Translation” that belongs to the high-level category “Protein synthesis”, Figure S5: The example of the upstream regulator network of CCN2., Figure S6: The example of the upstream regulator network of IL-10., Figure S7: The example of the upstream regulator network for the inhibited STAT1 in Epi C vs. Ctr group. The predicted inhibition of this putative upstream regulator relates to downregulation of selected chemokines and cytokines. The central term “STAT1” is filled with blue. A blue filling marks a predicted “decrease” of the function or process. The nodes in the periphery are filled with either green (downregulation in the experiment) or red (upregulation in the experiment), Figure S8: The first relevant and significant canonical pathway for VA vs. Ctr treatment group is the “Osteoarthritis signaling”, Figure S9: The canonical pathway “HMGB1 signaling” overlaid with experimental data from VA vs. Ctr group, Figure S10: The first relevant and significant canonical pathway for Epi C vs. Ctr treatment group is the “Osteoarthritis signaling, Figure S11: The canonical pathway “mTOR signaling”, Table S1: Differentially expressed genes after RNA Sequencing, Table S2: Low-level (specific) process or functions that belong to the high-level category “Connective Tissue Disorder”, Table S3: The results of the Upstream Regulator Analysis filtered for compounds and drugs and sorted according to their predicted activity and the number of targets. For the VA vs Ctr treatment group, several anti-inflammatory or immunosuppressive chemicals, e.g. dexamethasone or glucocorticoid, were found to be on top of this list. Moreover, different small molecule compounds, known to interfere with the activity of MEK/MAP or PI3 kinases, were also found to be high in that list of putative Upstream Regulators, Table S4: The results of the Upstream Regulator Analysis filtered for compounds and drugs and sorted according to their predicted activity and the number of targets. For the Epi C vs Ctr treatment group, several anti-inflammatory or immunosuppressive chemicals, e.g. dexamethasone or glucocorticoid, were found to be on top of this list. All z scores are above 2 which showed they are significantly activated.
