# Supplementary material for: Anti-Inflammatory and Chondroprotective Effects of Vanillic Acid and Epimedin C in Human Osteoarthritic Chondrocytes
Source: Biomolecules. 2020 Jun 19;10(6):932. doi: 10.3390/biom10060932 (PMC7356262; doi:10.3390/biom10060932)
Supplement: Supplementary file 1 [file biomolecules-10-00932-s001.pdf]

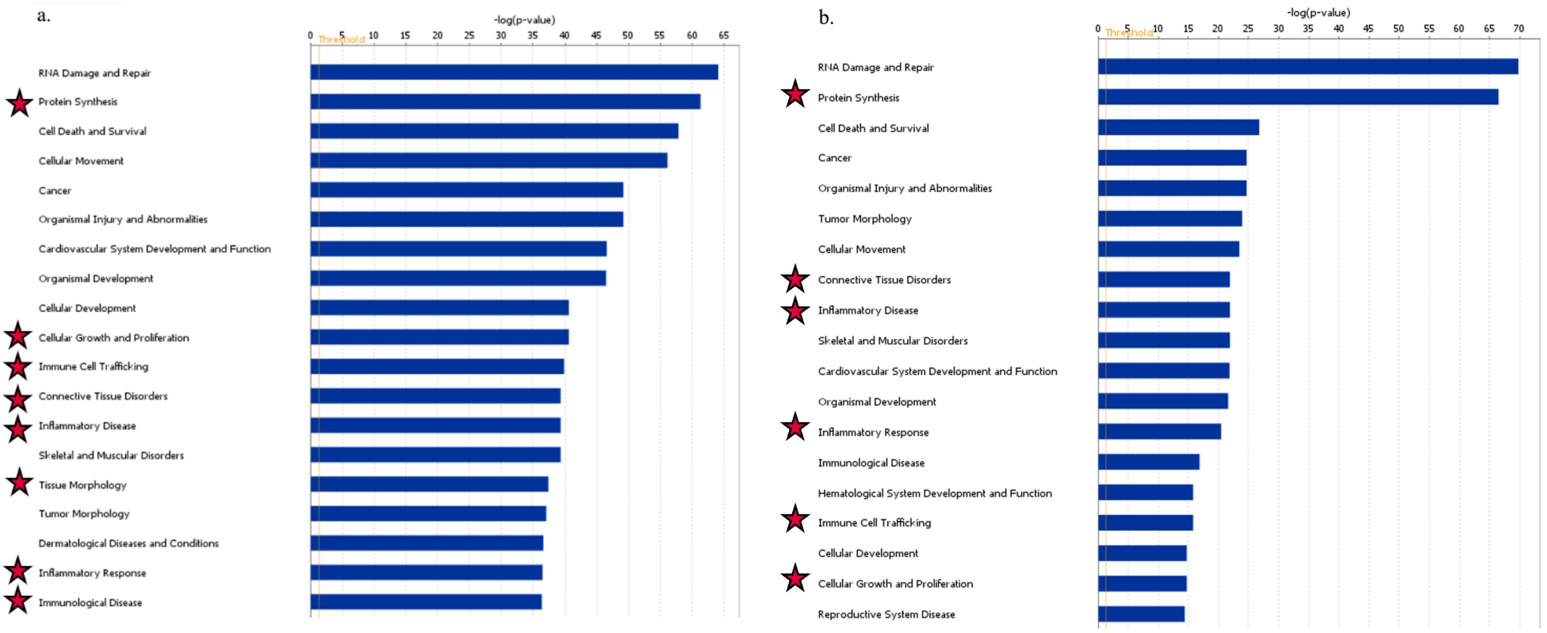

Figure S1. Top Diseases and Biological Functions from the Core analysis. **a**) Top results from the downstream effect analysis that was run as part of the Core analysis to identify functional endpoints, biological and cellular processes that overlap with the VA vs. Ctr negative dataset. **b**) Downstream effect analysis related to the biological and cellular processes that overlap with the Epi C vs. Ctr negative group dataset. The marked terms are high-level categories of biological and clinical processes or functions that fit to the expected biological context. The *p*-values are provided by Fisher's exact test (right tailed) to assess whether a given overlap is due to chance alone (low *p*-value rejects the null hypothesis = significant association).

a.

The Filter found 36 Functions for Immune Cell Trafficking

Categories	Diseases or Functions Annotation	p-value	Predicted Activation State	Activation z-score	Molecules	# Molecules
Cell-To-Cell Signali...	Activation of leukocytes	1.90E-18	Decreased	-3.828	↓AGRN, ↑AHNAK, ↑ANXA1, ↑AN... ...all 105	105
Cell-To-Cell Signali...	Activation of lymphocytes	5.46E-13	Decreased	-3.689	↓AGRN, ↑AHNAK, ↑ANXA1, ↓BTN... ...all 69	69
Cellular Movement,...	Migration of phagocytes	6.23E-18	Decreased	-3.447	↓ABCC1, ↓ABL1, ↑ALCAM, ↑ANXA1, ...all 59	59
Cellular Movement,...	Chemotaxis of leukocytes	9.23E-16	Decreased	-3.378	↓ACKR3, ↑ANXA1, ↓AQP9, ↓C3AR1, ...all 64	64
Cellular Movement,...	Chemotaxis of phagocytes	2.36E-15	Decreased	-3.335	↑ANXA1, ↓AQP9, ↓C3AR1, ↑C5AR2, ...all 56	56
Hematological Syst...	Accumulation of phagocytes	1.43E-18	Decreased	-3.313	↓ACKR3, ↑ANXA1, ↓CCL11, ↓CCL2, ...all 41	41
Cell-To-Cell Signali...	Activation of phagocytes	3.64E-13	Decreased	-3.100	↑ANXA1, ↑ANXA2, ↓ATF3, ↓BDNF, ...all 60	60
Hematological Syst...	Accumulation of leukocytes	1.15E-23	Decreased	-3.092	↓ACKR3, ↑ANXA1, ↓BCL2A1, ↑BGN, ...all 65	65
Cellular Movement,...	Cell movement of phagocytes	2.98E-26	Decreased	-2.970	↓ABCC1, ↓ABL1, ↓ADM, ↑ALCAM, ...all 110	110
Cellular Movement,...	Cell movement of macrophages	2.57E-19	Decreased	-2.850	↓ABL1, ↑ANXA1, ↓C3AR1, ↑CAV1, ...all 62	62
Cell-To-Cell Signali...	Activation of antigen presenting cells	5.27E-13	Decreased	-2.732	↑ANXA2, ↓ATF3, ↓BDNF, ↓CCL11, ...all 54	54
Cellular Movement,...	Homing of leukocytes	2.52E-18	Decreased	-2.716	↓ACKR3, ↑ANXA1, ↓AQP9, ↓C3AR1, ...all 71	71
Cellular Movement,...	Cell movement of neutrophils	5.02E-21	Decreased	-2.670	↓ADM, ↑ANXA1, ↑ANXA2, ↓AQP9, ...all 68	68
Cellular Movement,...	Cell movement of antigen presenting cells	4.69E-20	Decreased	-2.585	↓ABCC1, ↓ABL1, ↑ALCAM, ↑ANXA1, ...all 74	74
Cellular Movement,...	Migration of mononuclear leukocytes	2.21E-20	Decreased	-2.444	↓ABCC1, ↓ACKR3, ↑ANXA1, ↑ANX... ...all 76	76
Cell-To-Cell Signali...	Recruitment of leukocytes	4.27E-25	Decreased	-2.415	↑ADGRG6, ↑ANXA1, ↓C3AR1, ↑C... ...all 75	75
Cell-To-Cell Signali...	Recruitment of phagocytes	8.33E-19	Decreased	-2.368	↑ADGRG6, ↑ANXA1, ↓C3AR1, ↑C... ...all 56	56
Hematological Syst...	Accumulation of granulocytes	1.88E-14	Decreased	-2.301	↑ANXA1, ↓BCL2A1, ↓CCL11, ↓CCL2, ...all 30	30
Cellular Movement,...	Cell movement of granulocytes	1.42E-23	Decreased	-2.297	↓ADM, ↑ANXA1, ↑ANXA2, ↓AQP9, ...all 81	81
Cell-To-Cell Signali...	Adhesion of immune cells	3.59E-17	Decreased	-2.263	↓ABL1, ↑ALCAM, ↑ANXA1, ↓BMPE... ...all 66	66
Cellular Movement,...	Lymphocyte migration	5.11E-17	Decreased	-2.252	↓ABCC1, ↓ACKR3, ↑ANXA1, ↑CAV1, ...all 67	67
Cellular Movement,...	Cell movement of leukocytes	1.42E-34	Decreased	-2.240	↓ABCC1, ↓ABL1, ↓ACKR3, ↓ADM, ...all 152	152
Cellular Movement,...	Leukocyte migration	1.36E-40	Decreased	-2.008	↓ABCC1, ↓ABL1, ↓ACKR3, ↑ADG... ...all 182	182

b.

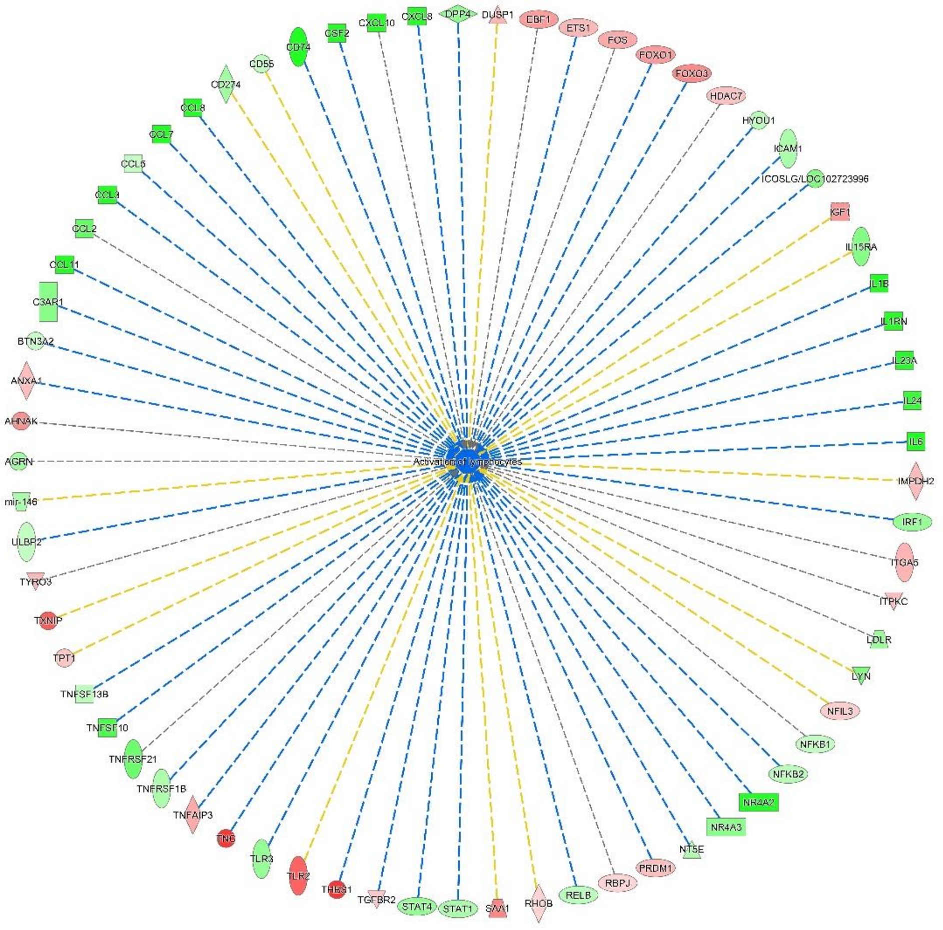

Figure S2. Low-level (specific) process or functions belonging to the high-level category "Immune cell trafficking". **a**) For the VA vs. Ctr group; most terms in this table have significantly negative activation z-scores. **b**) The network of the term "Activation of lymphocytes" in the list of top disorder in the high-level category "Immune cell trafficking". The central term "Activation of lymphocytes" is filled with blue. A blue filling marks a predicted "decrease" of the function or process. The nodes in

the periphery are filled with either green (down-regulation in the experiment) or red (up-regulation in the experiment).

a.

The Filter found 11 Functions for Inflammatory Disease

Categories	Diseases or Functions Annotation	p-value	Predicted Activation State	Activation z-score	Molecules	# Molecules
Connective Tissue Disorders,Inflamm...	Inflammation of joint	3.69E-21		-1.773	↑ACAN, ↑ADAMTS1, ↑ADGRG6, ↑ADRA... ...all 78	78
Connective Tissue Disorders,Inflamm...	Rheumatic Disease	1.28E-22		-1.134	↑ACAN, ↓ACSL5, ↑ADAMTS1, ↑ADGRG6, ...all 92	92
Inflammatory Disease	Chronic inflammatory disorder	4.03E-12		-0.651	↑ACAN, ↑ADGRG6, ↑ADRA2A, ↓AQP9, ↑... ...all 66	66
Connective Tissue Disorders,Inflamm...	Polyarthritis	2.02E-06		-0.577	↓CCL20, ↓CD74, ↑COL2A1, ↓CSF3, ↑GA... ...all 15	15
Connective Tissue Disorders,Immun...	Rheumatoid arthritis	1.76E-16			↑ACAN, ↑ADGRG6, ↑ADRA2A, ↓AQP9, ↑... ...all 58	58
Connective Tissue Disorders,Inflamm...	Osteoarthritis	4.63E-11			↑ACAN, ↑ADAMTS1, ↑ADRA2A, ↑ASPN, ...all 24	24
Inflammatory Disease,Neurological D...	Inflammatory demyelinating disease	4.45E-08			↑ADRA2A, ↑ALCAM, ↓BST2, ↓CCL2, ↑CR... ...all 21	21
Connective Tissue Disorders,Immun...	Systemic lupus erythematosus	8.59E-08			↓ACSL5, ↓BST2, ↓CCL2, ↓CXCL10, ↓EPS... ...all 26	26
Inflammatory Disease,Neurological D...	Multiple Sclerosis	1.53E-07			↑ADRA2A, ↑ALCAM, ↓BST2, ↓CCL2, ↑CR... ...all 20	20
Connective Tissue Disorders,Immun...	Polyarticular juvenile rheumatoid arth	6.51E-07			↓CCL20, ↓CD74, ↑GADD45B, ↑IL6R, ↓N... ...all 11	11
Connective Tissue Disorders,Inflamm...	Intervertebral disc disorder	4.10E-06			↑ADRA2A, ↑ASPN, ↑CCN2, ↑CILP, ↑COL1... ...all 8	8

b.

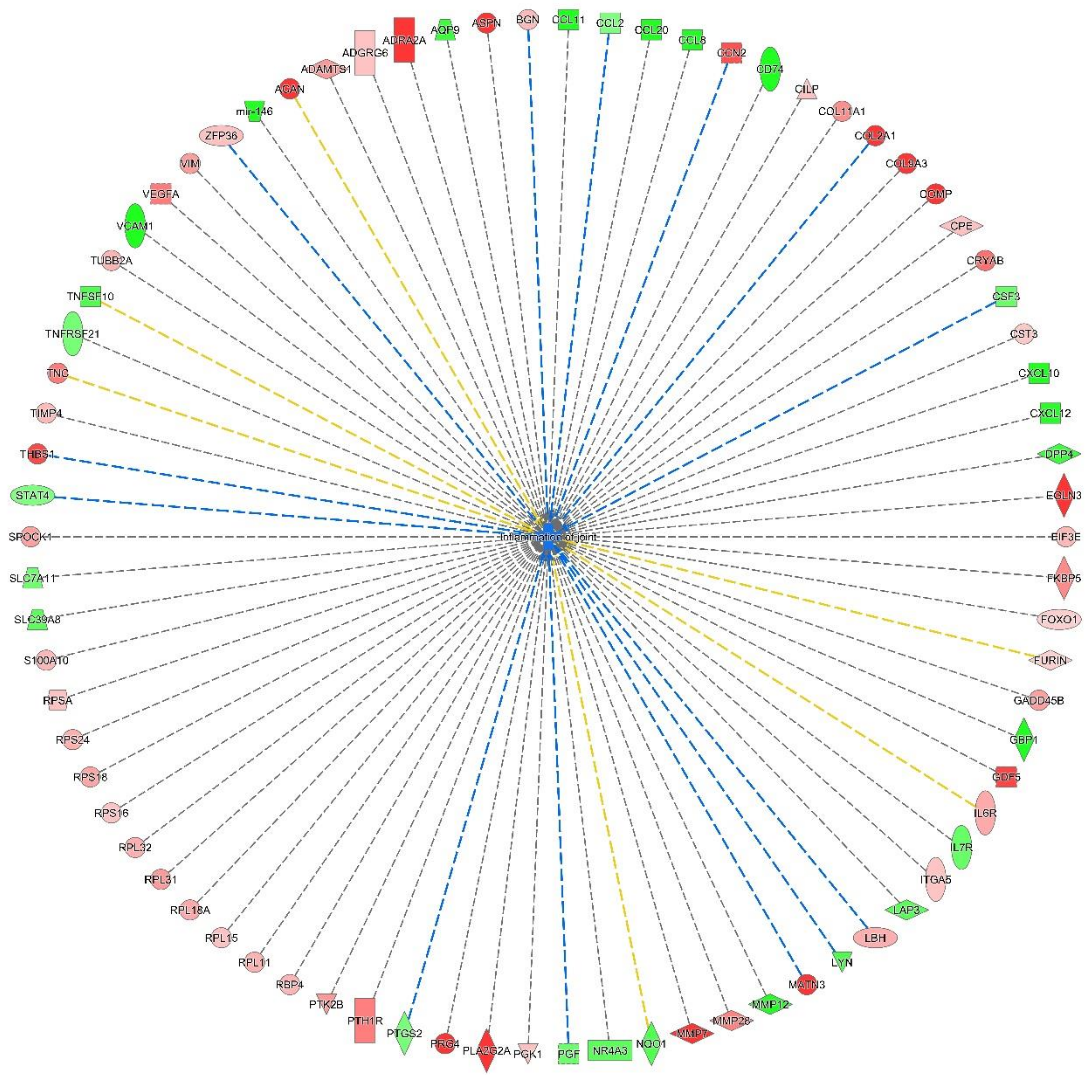

Figure S3. The example network of the term "Inflammation of the joints" **a**) the table of low-level (specific) process or functions that belong to the high-level category "Inflammatory disease" for Epi C vs. Ctr vehicle group. **b**) The term "Inflammation of the joints" was decreased and this is supported by down-regulated chemokines and cytokines as well as up-regulated ECM components, such as COL2A1, BGN, and CCN2. The central term "Inflammation of the joints" is filled with blue. A blue filling marks a predicted "decrease" of the function or process. The nodes in the periphery are filled with either green (down-regulation in the experiment) or red (up-regulation in the experiment).

a.

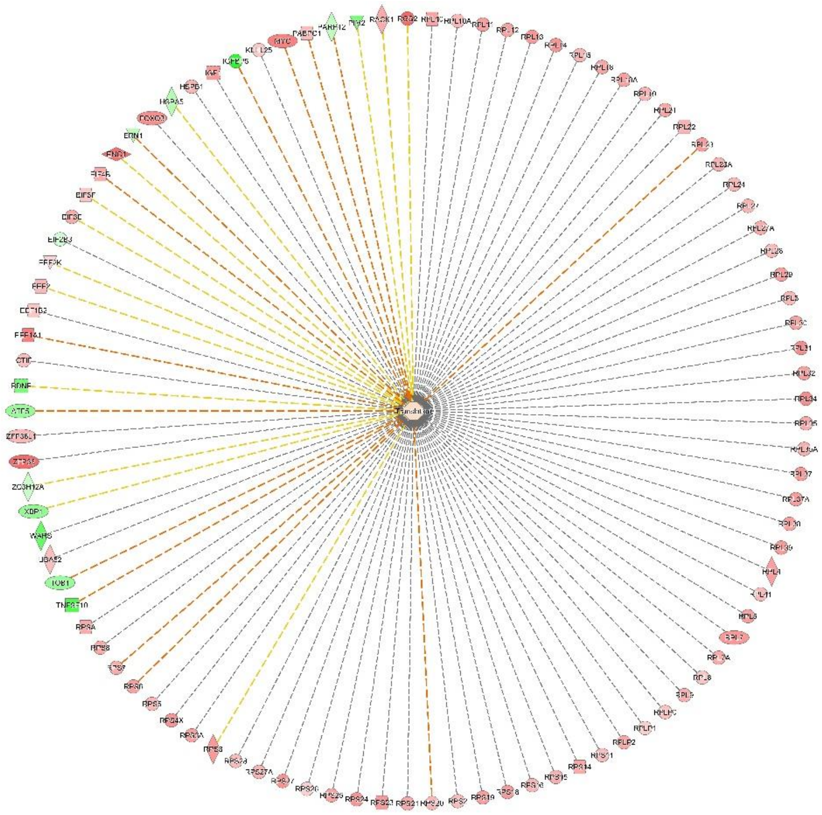

b.

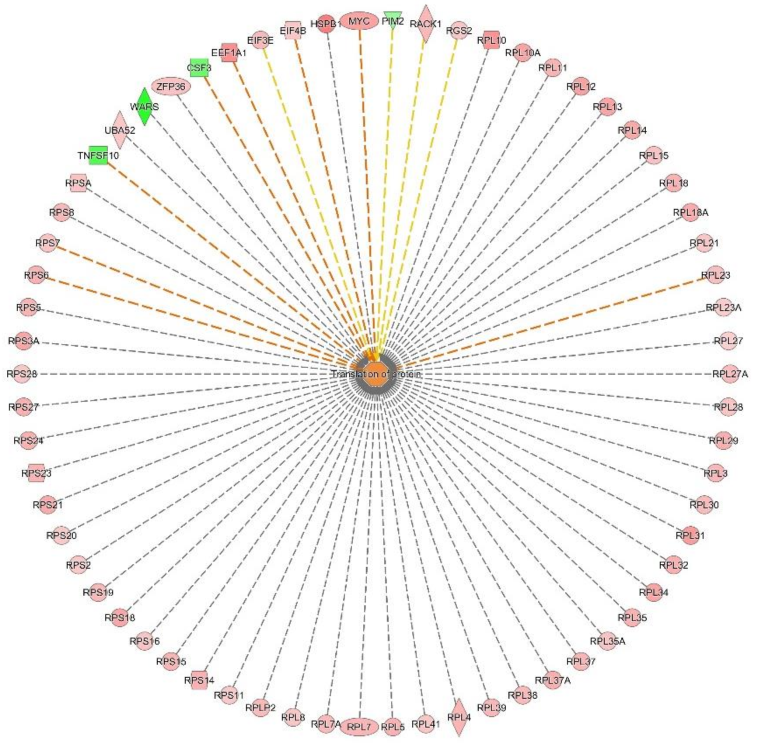

Figure S4. Example network of the term "Translation" that belongs to the high-level category "Protein synthesis". Most of these data set nodes represent ribosomal genes or translation initiation/elongation factors. **a**) The network for the VA vs. Ctr vehicle group and **b**) The network for the Epi C vs. Ctr

vehicle group are shown. The central node represents the term and the nodes in the periphery, associated with this term are shown. The central term "Translation" is filled with orange, which indicates the predicted tendency for "increase" (non-significant). The nodes in the periphery are filled with either green (down-regulation in the experiment) or red (up-regulation in the experiment). Some of the proteins with increased expression are COL5A1, COL6A3, COL8A1, BGN, LAMA2, LAMA3, LAMB1, LAMC1.

a.

b.

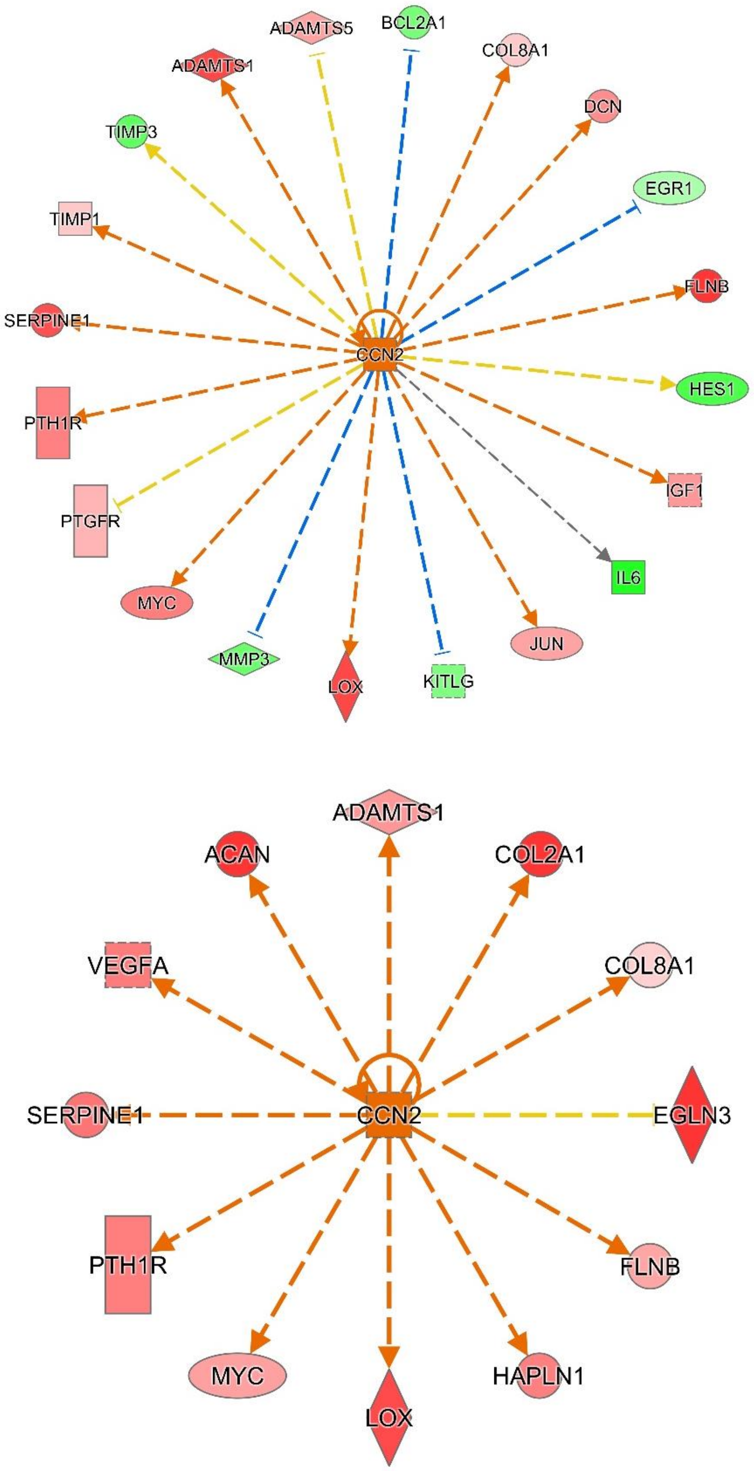

Figure S5. The example of the upstream regulator network of CCN2. **a**) The network for the VA vs. Ctr vehicle group and **b**) The network for the Epi C vs. Ctr vehicle group are shown. The central node

represents the upstream regulator and the nodes in the periphery data set represent molecules regulated by the upstream regulator. The central term “CCN2” is filled with orange, which indicates the predicted increase. The nodes in the periphery are filled with either green (down-regulation in the experiment) or red (up-regulation in the experiment). This network provides one explanation for the up-regulation of many ECM components.

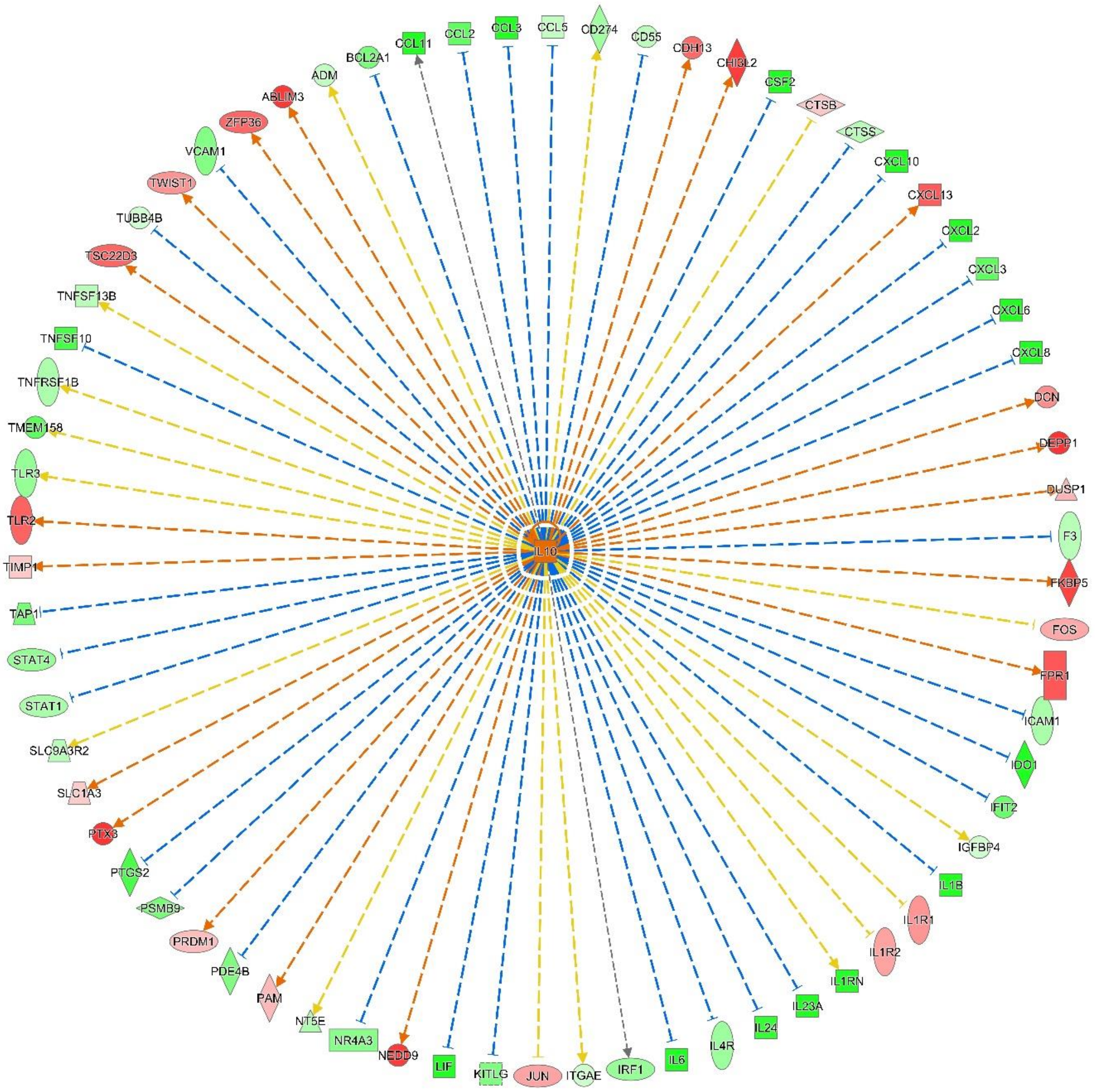

Figure 6. The example of the upstream regulator network of IL-10. The central node represents the upstream regulator and the nodes in the periphery data set represent molecules regulated by the upstream regulator. The central term "IL10" is filled with orange, which indicates the up-regulation of the IL-10. This network provides one explanation for the down-regulation of many different chemokines and cytokines in the experiment.

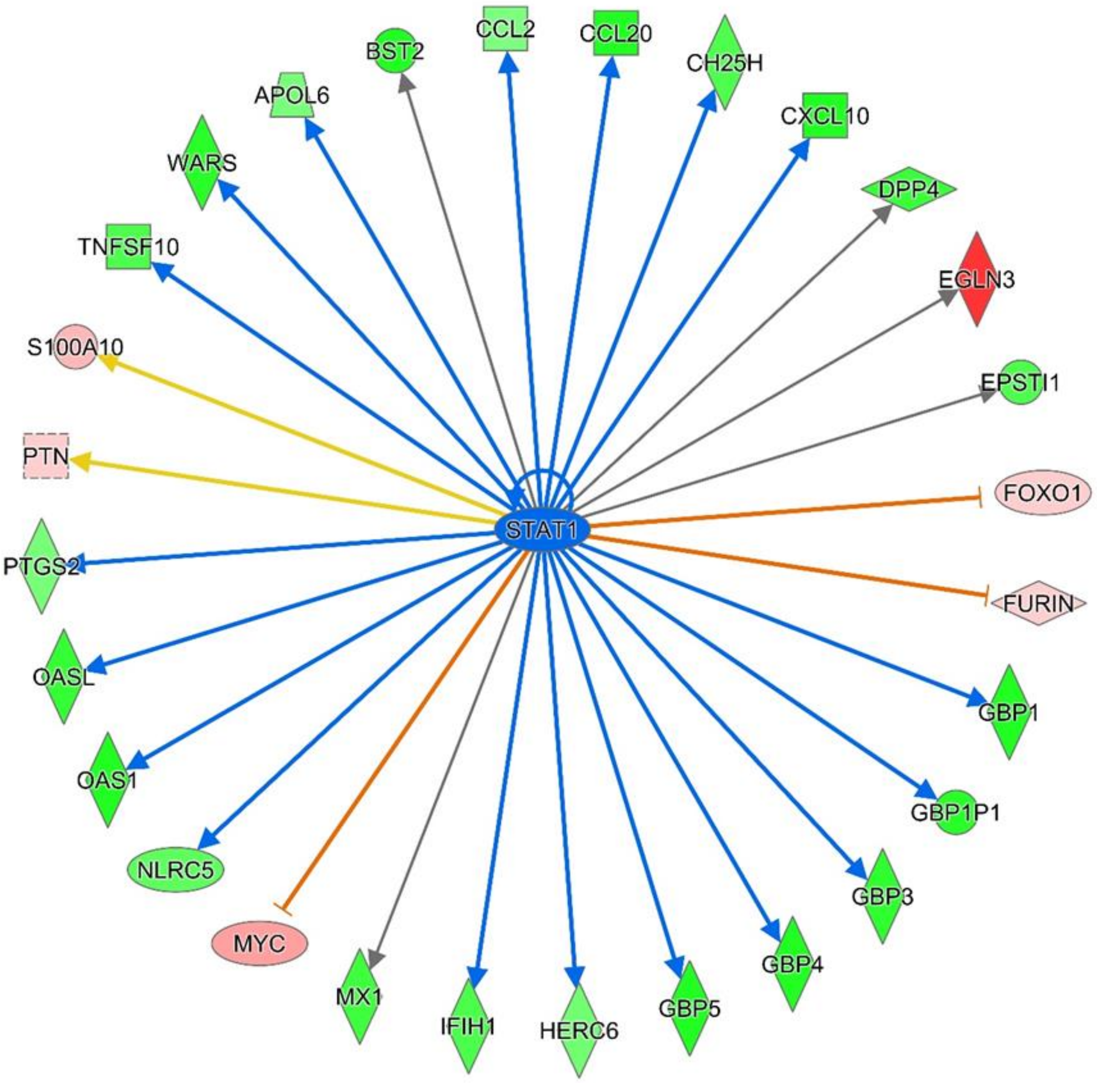

Figure S7. The example of the upstream regulator network for the inhibited STAT1 in Epi C vs. Ctr group. The predicted inhibition of this putative upstream regulator relates to down-regulation of selected chemokines and cytokines. The central term "STAT1" is filled with blue. A blue filling marks a predicted "decrease" of the function or process. The nodes in the periphery are filled with either green (down-regulation in the experiment) or red (up-regulation in the experiment).

a. b.

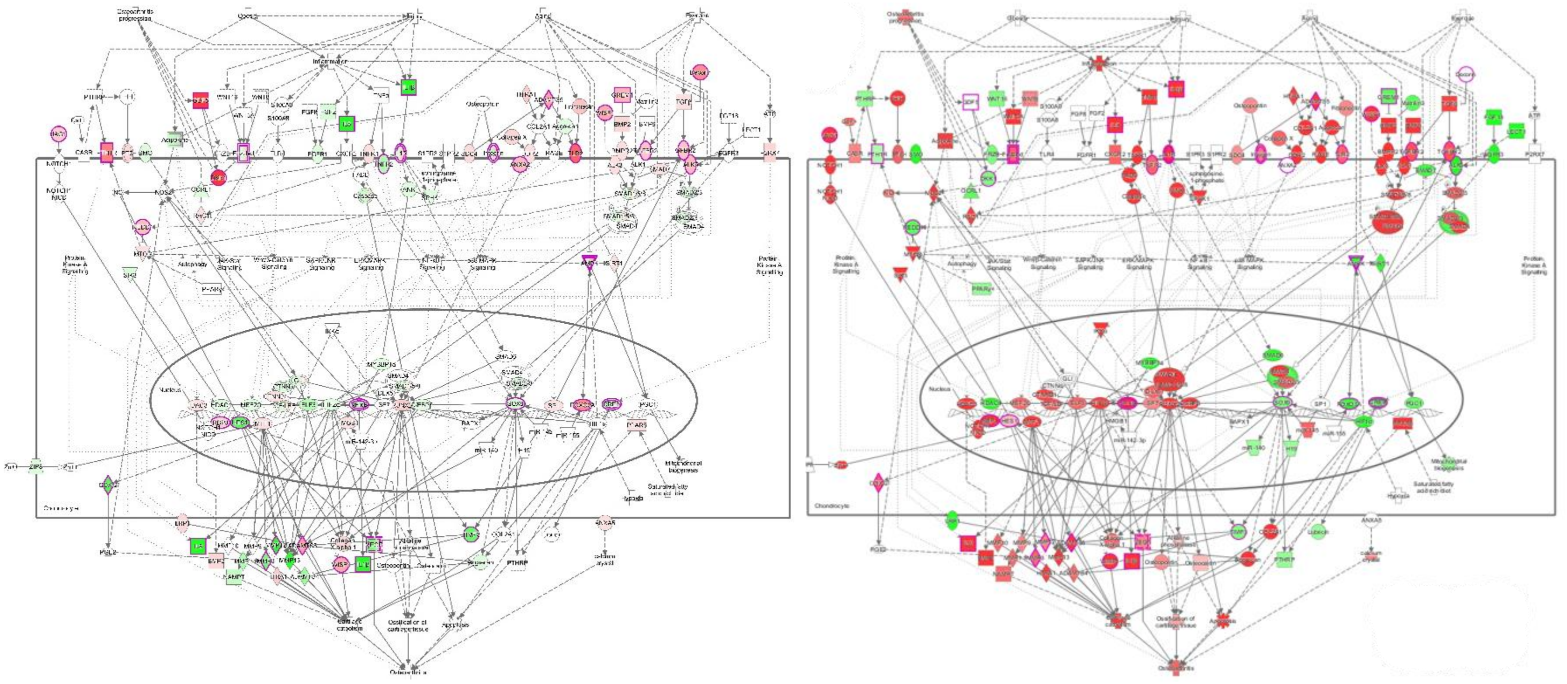

Figure S8. The first relevant and significant canonical pathway for VA vs. Ctr treatment group is the “Osteoarthritis signaling”. **a**) The activation z-score suggests a tendency for inhibition. Based on the activation z-score, the normal function of this pathway is reduced. Elements of this canonical pathway are shown as different shapes (nodes). These shapes are filled with red (up-regulation in the experiment) or green (down-regulation in the experiment). **b**) Shows the canonical pathway “Osteoarthritis signaling” overlaid with expected node activities considering this pathway as being fully activated (The expected activities are a result of curations of relevant papers, reviews and textbooks and can be compared to overlays of actual experimental measurements). Red fillings represent “activated”, green fillings “inhibited”. Nodes with purple highlights (borders) have passed the cut-offs.

a.

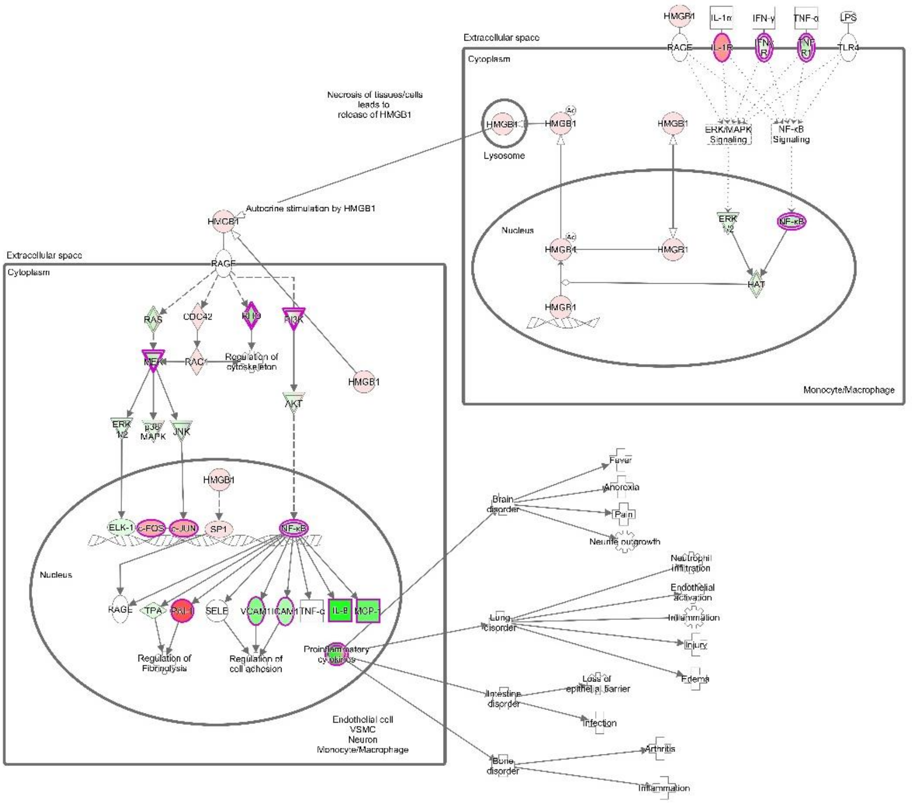

b.

b.

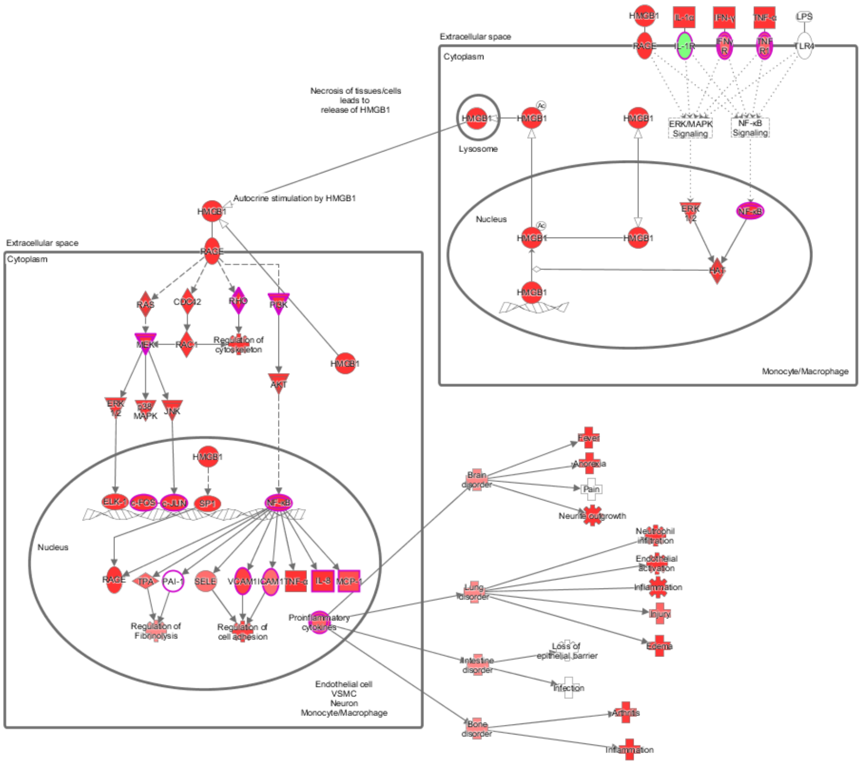

Figure S9. The canonical pathway "HMGB1 signaling" overlaid with experimental data from VA vs. Ctr group. **a**) The activation z-score is negative and is very close to the significance threshold. Therefore, we would assume that this pathway has a strong tendency to be inhibited. Central to this pathway is the down-regulation of NF-κB. **b**) "HMGB1 signaling" overlaid with expected activities. The comparison of the expected activities and actual measurements confirms the inhibition of this pathway. Red fillings represent "activated", green fillings "inhibited" purple highlights = passed cut-offs, no highlight = didn't pass cut-offs.

a. b.

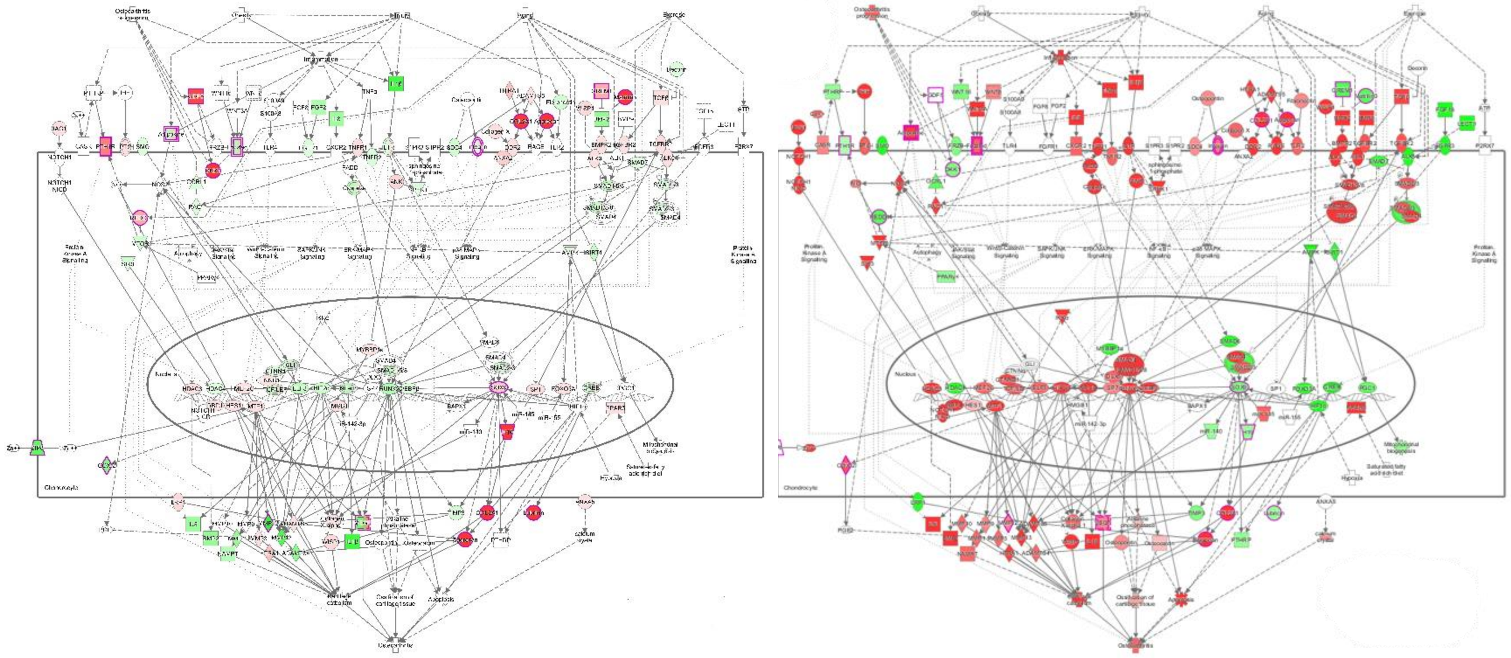

Figure S10. The first relevant and significant canonical pathway for Epi C vs. Ctr treatment group is the “Osteoarthritis signaling”. **a**) The activation *z*-score suggests a tendency for inhibition. Based on the activation *z*-score (*p*-value = 1.45E-3, *z*-score = -1.213), the normal function of this pathway is reduced. Elements of this canonical pathway are shown as different shapes (nodes). These shapes are filled with red (up-regulation in the experiment) or green (down-regulation in the experiment). **b**) Shows the canonical pathway “Osteoarthritis signaling” overlaid with expected node activities considering this pathway as being fully activated (The expected activities are a result of curations of relevant papers, reviews and textbooks and can be compared to overlays of actual experimental measurements). Red fillings represent “activated”, green fillings “inhibited”. Nodes with purple highlights (borders) have passed the cut-offs.

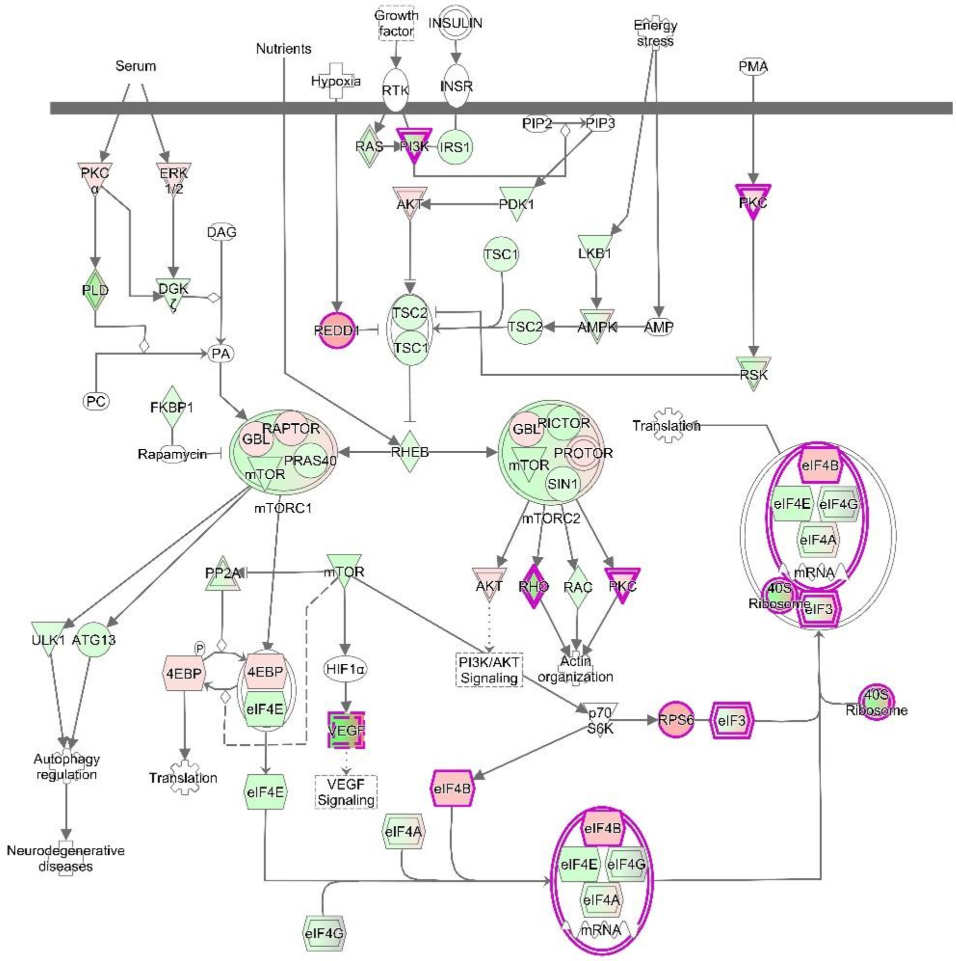

Figure S11. The canonical pathway "mTOR signaling". This pathway was found to be moderately activated (insignificant). A visual inspection of the pathway reveals up-regulated translation initiation/elongation factors and ribosomal genes. PKC and REDD1 are up-regulated. Red fillings represent "activated", green fillings "inhibited". Nodes with purple highlights (borders) have passed the cut-offs.

Table S1. Differentially expressed genes after RNA Sequencing.

5-HMF

Gene	Control Vehicle	5-HMF	log2_fold_change	q_value
TMC5	2.64693	0.0956953	-4.78973	0.0183303
CCL11	21.6693	1.02219	-4.40592	0.0183303
SLAMF7	3.7777	0.440358	-3.10076	0.0183303
LCNL1,PTGDS	32.3785	4.34702	-2.89694	0.0183303
RPLP0P2	13.0693	1.86573	-2.80837	0.0183303
IGFBP3	76.5181	11.124	-2.78213	0.0183303
LGI2	1.67212	0.243663	-2.77872	0.0183303
MEST	1.33042	0.220132	-2.59545	0.0183303
C6orf229,FAM65B	5.5852	0.951031	-2.55405	0.0183303
ADAMTS16	1.72847	0.304215	-2.50634	0.0424614
PLXDC1	9.36292	1.83855	-2.34839	0.0183303
BHLHE22	1.44655	0.313663	-2.20533	0.0424614
SYT7	3.87622	0.858705	-2.17441	0.0183303
MT1G	129.101	578.799	2.16456	0.0183303
GJB2	19.5447	4.45863	-2.13211	0.0183303
RANBP3L	2.02446	0.489476	-2.04823	0.0183303
CXCL12	44.7552	10.8482	-2.0446	0.0183303
COL14A1	5.90115	1.45538	-2.0196	0.0183303
RP11-231C14.6,SNX29P2	3.29369	0.834324	-1.98103	0.0183303
TNFSF18	1.85544	0.481394	-1.94647	0.0319471
CILP	8.55279	2.27515	-1.91043	0.0183303
C2CD4A	1.47128	0.393059	-1.90426	0.0424614
MT1H	43.8177	162.402	1.88999	0.0183303
SEMA3A	1.39115	0.378459	-1.87807	0.0183303
ARHGAP28	5.13577	1.43949	-1.83503	0.0183303
C3AR1	31.9442	9.50822	-1.74831	0.0183303
RP11-274B21.1	5.09561	1.53344	-1.73248	0.0183303
ADAMTS12	2.90448	0.884544	-1.71527	0.0183303
TMEM106A	1.71029	0.527054	-1.69822	0.0183303
CXCL10	44.7117	14.1341	-1.66147	0.0424614
SEMA5A	5.2664	1.70523	-1.62685	0.0183303
IGF1	3.39587	1.10182	-1.62389	0.0183303
ITGB8	6.63335	2.158	-1.62004	0.0183303
CPXM1	4.08831	1.3494	-1.59919	0.0183303
IL7R	10.756	3.59456	-1.58125	0.0183303
TRIM56	13.0652	4.39521	-1.57172	0.0183303
-	2.15229	0.727996	-1.56387	0.0183303
FOXP2	1.57725	0.537849	-1.55214	0.0183303
IGFBP5	81.9929	28.4864	-1.52523	0.0183303
VCAM1	15.7227	5.48587	-1.51906	0.0183303
CH25H	91.9764	32.2598	-1.51153	0.0183303
SEMA4D	5.85942	2.06433	-1.50509	0.0183303
RP11-640M9.2	11.5303	4.09642	-1.49299	0.0183303

4-HBA

Gene	Control Vehicle	4-HBA	log2_fold_change	q_value
FOSB	0.263976	37.3909	7.14614	0.0155513
FOS	4.55402	144.096	4.98375	0.0155513

CCL11	21.8856	1.00344	-4.44696	0.0155513
-	0.236927	4.01346	4.08233	0.0351059
C2CD4A	1.482	0.132841	-3.47978	0.0155513
CXCL12	45.0349	4.5926	-3.29366	0.0155513
MMP12	14.236	1.65481	-3.1048	0.0155513
C6orf229,FAM65B	5.60808	0.693199	-3.01617	0.0155513
ADAMTS16	1.7401	0.225879	-2.94555	0.0155513
CXCL13	290.288	40.0756	-2.85669	0.0155513
SFRP2	14.832	2.04785	-2.85653	0.0155513
-	2.65095	0.370007	-2.84088	0.0155513
EGR1	20.5017	142.705	2.79922	0.0155513
RPLP0P2	13.1534	2.08821	-2.6551	0.0155513
COL14A1	5.93573	0.951672	-2.64089	0.0155513
IGFBP3	77.005	12.7427	-2.59528	0.0155513
GJB2	19.6628	3.41116	-2.52714	0.0155513
RP11-367F23.2	1.72502	0.301614	-2.51583	0.0446
CADM3,DARC	5.25611	0.931491	-2.49638	0.0155513
SYT7	3.90202	0.710104	-2.45812	0.0155513
MMP8	24.1625	4.40359	-2.45602	0.0155513
PLXDC1	9.42433	1.91707	-2.29749	0.0155513
AP001434.2, SPATA20P1	3.33822	0.690853	-2.27263	0.0268614
CD248	12.3031	2.87461	-2.09759	0.0155513
QPRT,SPN	2.53519	0.595588	-2.08971	0.0155513
NCALD	1.51106	0.361393	-2.06392	0.0155513
CPXM1	4.1156	1.02489	-2.00563	0.0155513
CH25H	92.5579	23.3474	-1.98709	0.0155513
ADAMTS12	2.92097	0.746958	-1.96735	0.0155513
HAS2	13.1636	3.43491	-1.93821	0.0155513
CILP	8.59468	2.31573	-1.89198	0.0155513
-	1.06049	0.29116	-1.86484	0.0351059
ARHGAP28	5.1644	1.46177	-1.82089	0.0155513
C3AR1	32.056	9.12723	-1.81235	0.0155513
KIAA0226L	6.41788	1.89241	-1.76187	0.0155513
TSPAN11	22.1212	6.53724	-1.75868	0.0155513
SFRP1	1.58589	0.472192	-1.74784	0.0351059
SEMA5A	5.26718	1.57554	-1.74119	0.0155513
IL7R	10.8159	3.26278	-1.72898	0.0155513
CSF2	8.06655	25.8906	1.6824	0.0155513
GALR3	0.488738	1.55064	1.66573	0.0155513
SERPINA1	12.5967	4.05062	-1.63683	0.0268614

Vanillic acid

Gene	Control Vehicle	VA	log2_fold_change	q_value
EIF1AY	12.2023	0.0277272	-8.78163	0.0104271
CCL11	20.913	0.203771	-6.6813	0.00330143
MMP12	13.6562	0.169591	-6.33135	0.00330143
CXCL10	43.1215	0.553861	-6.28274	0.00330143
IDO1	6.66431	0.122341	-5.76748	0.00330143
IL23A	182.688	3.8186	-5.58019	0.00330143
MMP7	2.47688	107.857	5.44446	0.00330143
RP11986E7.7,SERPI3, SERPINA4,SERPIN5	10.8106	405.536	5.22931	0.00330143
HIF3A	0.514046	16.8623	5.03576	0.00330143
IL6	6709.62	205.125	-5.03165	0.00330143

LAMA3	0.695779	21.194	4.92888	0.00330143
DKK1	0.913079	26.3309	4.84988	0.00330143
APOD	3.15709	80.1754	4.66649	0.00330143
EREG	58.573	2.53097	-4.53247	0.00330143
SLCO2B1	4.068	0.180563	-4.49374	0.00330143
PTX3	64.4246	1443.97	4.48629	0.00330143
DCLK1	0.0972317	2.08802	4.42457	0.00330143
LINC00473	12.0236	0.64595	-4.2183	0.00330143
CCL8	25.3413	1.41583	-4.16178	0.00330143
PRELP	2.75364	47.4012	4.10551	0.00330143
ZBED3	0.244914	4.19019	4.09667	0.00330143
ITGBL1	10.8984	183.9	4.07674	0.00330143
GBP5	6.94778	0.423924	-4.03467	0.00330143
SLC38A4	0.231164	3.77807	4.03066	0.00330143
PRODH	0.169971	2.54284	3.90308	0.0124428
THBS1	17.9431	259.742	3.85558	0.00330143
TMEM150C	0.840441	11.3287	3.75269	0.00330143
TJP2	1.30083	17.5244	3.75186	0.00330143
FAM46B	0.114869	1.5271	3.73274	0.00330143
CCL3	8.1225	0.623825	-3.70271	0.00330143
ADH1B	0.316882	4.08275	3.68752	0.00330143
IL11	350.332	28.4794	-3.62073	0.00330143
CYP4F22	0.174107	2.13505	3.61622	0.00330143
NCAM1	0.0925037	1.11978	3.59757	0.00330143
CTD-2218G20.1	0.205667	2.37841	3.53161	0.00592391
TNFSF18	1.79154	0.158897	-3.49504	0.00330143
ST6GALNAC5	0.238698	2.5881	3.43863	0.00330143
GPR98,LUCAT1,RP11-213H15.4	2.4874	0.24385	-3.35057	0.0104271
FGFBP2,PROM1	0.431683	4.33999	3.32965	0.00330143
RP11-855A2.5	4.77143	0.496706	-3.26396	0.0416914
COMP	29.7354	272.884	3.19803	0.00330143
SERPINB3,SERPINB4	22.8248	2.52288	-3.17746	0.00330143
ADRA2A	1.29417	11.654	3.17073	0.00330143

Psoralidin

Gene	Control Vehicle	PS	log2_fold_change	q_value
TMC5	2.63217	0.0815934	-5.01166	0.0296
GPR171	1.83483	0.10289	-4.15647	0.0471184
CCL11	21.5156	1.50912	-3.8336	0.0296
SLAMF7	3.75147	0.384534	-3.28627	0.0296
MMP12	14.0629	1.64358	-3.09698	0.0296
IGFBP3	75.9714	10.1393	-2.9055	0.0296
C6orf229,FAM65B	5.54657	0.758774	-2.86985	0.0296
CXCL12	44.4236	7.68137	-2.53189	0.0296
ADAMTS16	1.71537	0.306635	-2.48393	0.0296
H19	0.547717	2.84943	2.37917	0.0471184
SFRP2	14.6784	2.89915	-2.33999	0.0296
-	2.61808	0.550791	-2.24893	0.0296
C2CD4A	1.46212	0.307976	-2.24717	0.0471184

COL14A1	5.8572	1.35211	-2.115	0.0296
COL5A3	7.20995	1.78696	-2.01249	0.0296
CD248	12.1363	3.0379	-1.99818	0.0296
OLFML2B	47.6949	12.0031	-1.99043	0.0296
CXCL13	287.107	78.1301	-1.87764	0.0296
GJB2	19.3988	5.29622	-1.87293	0.0296
CXCL10	44.4021	12.2553	-1.85722	0.0296
QPRT,SPN	2.50082	0.707217	-1.82218	0.0296
PLXDC1	9.29195	2.85854	-1.7007	0.0296
SERPING1	21.0017	6.4938	-1.69337	0.0296
SYT7	3.84399	1.20596	-1.67242	0.0296
CILP	8.48368	2.78906	-1.60491	0.0296
RASD1	8.76984	2.92944	-1.58192	0.0296
RPLP0P2	12.9542	4.35935	-1.57123	0.0471184
MAPK10	4.61556	1.57044	-1.55534	0.0296
ARHGAP28	5.09554	1.78527	-1.51309	0.0296
VCAM1	15.6117	5.47361	-1.51206	0.0296
S100A4	517.472	184.818	-1.48537	0.0296
TSPAN11	21.8221	8.40774	-1.376	0.0296
RP11-82L18.4	1.74237	0.711546	-1.29202	0.0471184
MMP8	23.8542	9.78617	-1.28543	0.0296
CH25H	91.3212	39.3359	-1.2151	0.0296
IL7R	10.6755	4.62532	-1.20668	0.0471184
MIR23A,MIR24-2,MIR27A	17.6043	7.74944	-1.18376	0.0471184
ITGB8	6.56586	3.00451	-1.12785	0.0296
ISLR	62.1145	29.6579	-1.06651	0.0296
GAS1	42.5655	20.8757	-1.02786	0.0296
SMOC1	238.548	119.825	-0.993346	0.0471184
HMOX1	197.539	392.255	0.989654	0.0296
IGFBP5	81.4198	43.0461	-0.919498	0.0296

PCA

Gene	Control Vehicle	PCA	log2_fold_change	q_value
CCL11	21.6406	0.56146	-5.26841	0.020294
MMP12	14.1281	0.580278	-4.60568	0.020294
IDO1	6.89688	0.348589	-4.30634	0.020294
CXCL10	44.6263	4.00811	-3.4769	0.020294
MIR4477A	0.883464	8.1207	3.20036	0.020294
LGI2	1.66828	0.21936	-2.92699	0.020294
RPLP0P2	13.0234	2.07816	-2.64773	0.020294
SFRP2	14.7299	2.44462	-2.59106	0.020294
CXCL12	44.6469	7.88936	-2.50058	0.020294
PDGFD	1.05835	0.18828	-2.49086	0.020294
-	1.60907	0.301419	-2.41638	0.020294
PLXDC1	9.3396	1.81885	-2.36034	0.020294
SYT7	3.864	0.815163	-2.24493	0.020294
ADAMTS12	2.8952	0.615334	-2.23422	0.020294
CD74	69.8431	15.3655	-2.18443	0.020294
IGFBP3	76.3365	17.9516	-2.08826	0.020294
-	2.63019	0.642128	-2.03423	0.0373667
RP11-81H14.2	5.93571	1.47732	-2.00644	0.0373667
MIR145	1.90096	0.475952	-1.99784	0.020294
SERPING1	21.1015	5.49679	-1.94069	0.020294
CH25H	91.7586	23.9356	-1.93868	0.020294

GJB2	19.4963	5.16469	-1.91645	0.020294
VCAM1	15.6878	4.15746	-1.91586	0.020294
COL14A1	5.88404	1.81486	-1.69695	0.020294
ARHGAP28	5.12052	1.61141	-1.66796	0.020294
EGR1	20.3424	63.7766	1.64854	0.020294
S100A4	519.91	166.012	-1.64697	0.020294
TMEM158	19.1705	6.14241	-1.64201	0.020294
LIPG	4.26873	1.39372	-1.61486	0.020294
TMEM119	64.4319	21.1053	-1.61017	0.020294
DPP4	11.0845	3.65438	-1.60084	0.020294
TSPAN11	21.9278	7.36666	-1.57368	0.020294
CD248	12.195	4.10483	-1.57089	0.020294
C3AR1	31.7871	10.8534	-1.5503	0.020294
CXCL13	288.38	102.208	-1.49646	0.020294
CDCP1	4.40174	1.58396	-1.47454	0.020294
ADAMTS14	3.95449	1.44174	-1.45568	0.020294
IL7R	10.7273	3.93439	-1.44707	0.020294
SEMA5A	5.2209	1.93812	-1.42964	0.020294
C9orf47,S1PR3	16.0296	6.10935	-1.39165	0.020294
HAS2	13.0453	5.02277	-1.37698	0.020294
ITGB8	6.59655	2.5618	-1.36455	0.020294

Epimedin C

Gene	Control Vehicle	Epi C	log2_fold_change	q_value
H19	0.537211	65.6371	6.93288	0.0182
MMP7	2.4939	194.161	6.2827	0.0182
HIF3A	0.5177	22.0082	5.40978	0.0182
CA9	2.0508	70.6185	5.10579	0.0383362
RAB11FIP4	0.159334	4.72276	4.88951	0.0182
SPARCL1	0.721847	17.0604	4.56281	0.0182
COL2A1	0.625102	14.7129	4.55684	0.0182
APOD	3.18023	69.3519	4.44673	0.0182
SLC38A4	0.232951	4.7077	4.33692	0.0182
RP11986E7.7,SERPINA3, SERPINA4, SERPINA5	10.8912	195.871	4.16867	0.0182
APLN	0.0956189	1.48885	3.96076	0.0182
COMP	29.9424	463.836	3.95335	0.0182
RSPO2	0.649701	9.99886	3.94392	0.0182
CAPN6	0.25947	3.93384	3.9223	0.0182
HSPB7	0.84741	12.8034	3.91732	0.0182
MATN3	1.41254	20.7226	3.87484	0.0182
DCLK1	0.0979673	1.39937	3.83633	0.0182
PRELP	2.77419	34.6352	3.6421	0.0182
RP11-434D9.1	0.2051	2.5318	3.62576	0.0182
DKK1	0.91998	10.587	3.52455	0.0490286
-	0.716545	7.77418	3.43956	0.0304101
ST6GALNAC5	0.240451	2.43521	3.34023	0.0182
SCRG1	20.5605	207.495	3.33513	0.0182
CTD-2561J22.2	0.255837	2.54356	3.31355	0.0304101
CLEC18A	0.249291	2.20312	3.14365	0.0182
ACAN	10.9287	94.5459	3.11289	0.0182
ZBED3	0.246552	2.06441	3.06577	0.0182
KIAA1456	0.835823	6.91398	3.04825	0.0182
ITGA10	5.30106	42.1127	2.9899	0.0182

FGFBP2,PROM1	0.43552	3.40952	2.96876	0.0182
MMP12	13.7658	1.82289	-2.91679	0.0383362
GPM6B	3.47194	25.5839	2.88142	0.0182
GBP5	7.00352	0.980563	-2.8364	0.0182
CDH23	1.15792	0.16522	-2.80908	0.0304101
ADRA2A	1.30369	9.11261	2.80527	0.0182
WWP2	23.0827	143.939	2.64058	0.0182
S100B	0.781904	4.80197	2.61856	0.0304101
GDF5	1.70156	10.2381	2.58902	0.0182
MFAP5	0.465845	2.73492	2.55358	0.0182
THBS1	18.0735	104.083	2.52579	0.0182
LRMP	1.25509	0.23105	-2.44151	0.0182
CTGF	21.7411	115.549	2.41	0.0182
-	0.349015	1.8206	2.38305	0.0490286
RP11-284F21.8	0.283548	1.47866	2.38262	0.0182
CADPS	0.211655	1.0837	2.35618	0.0182

Table S2. Low-level (specific) process or functions that belong to the high-level category "Connective Tissue Disorder". **a**) For the VA vs Ctr group, 4 terms including Inflammation of the joint, Rheumatic disease, experimentally induced arthritis, polyarthritis with significant activation z-scores indicate a significant decrease of all these functions. **b**) For the Epi C vs Ctr group abnormality of cartilage tissue, Inflammation of joint, are top results with negative z-scores (predicted decrease, non-significant).

a.

The Filter found 10 Functions for Connective Tissue Disorders

Categories	Diseases or Functions Annotation	p-value	Predicted Activation State	Activation z-score	Molecules	# Molecules
Connective Tissue D...	Inflammation of joint	3.31E-37	Decreased	-2.678	↓ABCC1, ↑ABCC2, ↓ABL1, ↑ADA... ...all 181	181
Connective Tissue D...	Rheumatic Disease	4.82E-40	Decreased	-2.501	↓ABCC1, ↑ABCC2, ↓ABL1, ↓ACSL5, ...all 216	216
Connective Tissue D...	Experimentally-induced arthritis	1.19E-13	Decreased	-2.151	↑ANXA1, ↑CDH11, ↓CTSS, ↑CXCL13, ...all 36	36
Connective Tissue D...	Polyarthritis	5.11E-17	Decreased	-2.133	↓ADM, ↑ANXA1, ↓CCL3, ↓CCL5, ↓... ...all 43	43
Connective Tissue D...	Systemic lupus erythematosus	1.72E-13		-0.614	↓ACSL5, ↓ADAMTS6, ↑ANXA1, ↓B... ...all 61	61
Connective Tissue D...	Lupus erythematosus	1.00E-13		-0.248	↓ACSL5, ↓ADAMTS6, ↑ANXA1, ↓B... ...all 63	63
Connective Tissue D...	Non-traumatic arthropathy	1.63E-35		-0.021	↓ABCC1, ↑ABCC2, ↑ADAMTS1, ↑... ...all 159	159
Connective Tissue D...	Rheumatoid arthritis	2.44E-29		0.129	↑ADAMTS2, ↑ADGRG6, ↓ADM, ↑... ...all 135	135
Connective Tissue D...	Osteoarthritis	3.38E-16		0.557	↑ADAMTS1, ↑ADAMTS2, ↑ADAMT... ...all 49	49
Connective Tissue D...	Polyarticular juvenile rheumatoid arthritis	1.76E-13			↓ADM, ↓CCL3, ↓CCL5, ↓CD274, ↓C... ...all 26	26

b.

The Filter found 19 Functions for Connective Tissue Disorders

Categories	Diseases or Functions Annotation	p-value	Predicted Activation State	Activation z-score	Molecules	# Molecules
Connective Tissue Disorders,Organis...	Abnormality of cartilage tissue	2.50E-08		-1.915	↑ACAN, ↑ADRA2A, ↑ASPN, ↑BGN, ↑CC... ...all 18	18
Connective Tissue Disorders,Inflam...	Inflammation of joint	3.69E-21		-1.773	↑ACAN, ↑ADAMTS1, ↑ADGRG6, ↑ADRA... ...all 78	78
Connective Tissue Disorders,Organis...	Non-traumatic arthropathy	1.73E-21		-1.446	↑ACAN, ↑ADAMTS1, ↑ADGRG6, ↑ADRA... ...all 71	71
Connective Tissue Disorders,Inflam...	Rheumatic Disease	1.28E-22		-1.134	↑ACAN, ↓ACSL5, ↑ADAMTS1, ↑ADGRG6, ...all 92	92
Connective Tissue Disorders,Heredit...	Hereditary connective tissue disorder	7.02E-07		-1.054	↑ACAN, ↑ADGRG6, ↑ASPN, ↑BGN, ↓CC... ...all 33	33
Connective Tissue Disorders,Develop...	Dysplasia of skeleton	7.97E-10		-1.000	↑ACAN, ↑ADRA2A, ↑BGN, ↑CCN2, ↑CO... ...all 21	21
Connective Tissue Disorders,Inflam...	Polyarthritis	2.02E-06		-0.577	↓CCL20, ↓CD74, ↑COL2A1, ↓CSF3, ↑GA... ...all 15	15
Connective Tissue Disorders,Immun...	Rheumatoid arthritis	1.76E-16			↑ACAN, ↑ADGRG6, ↑ADRA2A, ↓AQP9, ↑... ...all 58	58
Connective Tissue Disorders,Inflam...	Osteoarthritis	4.63E-11			↑ACAN, ↑ADAMTS1, ↑ADRA2A, ↑ASPN, ...all 24	24
Connective Tissue Disorders,Develop...	Familial skeletal dysplasia	7.78E-09			↑ACAN, ↑BGN, ↑CCN2, ↑COL11A1, ↑CO... ...all 19	19
Connective Tissue Disorders,Develop...	Chondrodysplasia	3.37E-08			↑ACAN, ↑BGN, ↑CCN2, ↑COL11A1, ↑CO... ...all 11	11

Table S3. The results of the Upstream Regulator Analysis filtered for compounds and drugs and sorted according to their predicted activity and the number of targets. For the VA vs Ctr treatment group, several anti-inflammatory or immunosuppressive chemicals, e.g. dexamethasone or glucocorticoid, were found to be on top of this list. Also, different small molecule compounds, known to interfere with the activity of MEK/MAP or PI3 kinases were also found to be high in that list of putative Upstream Regulators.

MEK/MAP;PI3K Inhibitors (bracketed: U0126, PD98059, LY294002, SB203580)

Upstream Regulator	Expr Log Ratio	Molecule Ty...	Predicted Ac...	Activation z...	p-value of o...	Target ...	Mechanistic...
dexamethasone		chemical drug	Activated	8.402	1.33E-85	↓ABCC1,↑.. ...all 298	573 (22)
U0126		chemical - kinase in...	Activated	3.282	1.47E-42	↓ACKR3,↓.. ...all 106	297 (13)
PD98059		chemical - kinase in...	Activated	2.421	9.49E-39	↑ABCC2,↑.. ...all 106	378 (14)
LY294002		chemical - kinase in...	Activated	2.354	3.67E-34	↓ABCC1,↓... ...all 96	388 (12)
SB203580		chemical - kinase in...	Activated	3.312	1.66E-34	↑ANXA1,↓......all 90	425 (15)
filgrastim		biologic drug	Activated	3.869	2.69E-23	↑ALDH6A1,·...all 73	
cyclosporin A		biologic drug	Activated	2.733	1.44E-16	↑ABCC2,↓... ...all 63	393 (18)
fluticasone propionate		chemical drug	Activated	4.613	2.53E-40	↑ABLIM3,↓......all 62	454 (18)
genistein		chemical drug	Activated	2.486	8.19E-15	↓ADM, ↓A... ...all 56	364 (15)
diethylstilbestrol		chemical drug	Activated	2.089	6.08E-16	↓AQP9, ↑B......all 50	481 (21)
glucocorticoid		chemical drug	Activated	3.721	4.69E-16	↑ANXA1, ↓......all 49	313 (14)
epigallocatechin-gallate		chemical drug	Activated	2.940	7.68E-15	↓CCL2, ↓C... ...all 41	314 (16)
15-deoxy-delta-12,14 -PGJ 2		chemical - endogen...	Activated	2.166	1.69E-14	↓BIRC3, ↓... ...all 36	292 (15)
simvastatin		chemical drug	Activated	2.109	1.07E-11	↑ADAMTS1, ...all 35	344 (15)
methotrexate		chemical drug	Activated	2.772	1.72E-09	↓CCL11, ↓... ...all 34	377 (17)
prednisolone		chemical drug	Activated	3.044	4.46E-10	↓BAG1, ↓B... ...all 34	419 (19)
bexarotene		chemical drug	Activated	2.753	2.59E-10	↓AKR1C3,↑.. ...all 31	487 (22)
caffeic acid phenethyl ester		chemical drug	Activated	2.652	3.26E-15	↓ABL1, ↓B... ...all 28	280 (12)
hydrocortisone		chemical - endogen...	Activated	3.050	3.32E-10	↓ADM, ↓C... ...all 26	463 (21)
Sb202190		chemical - kinase in...	Activated	2.657	7.96E-10	↓CCL2, ↓C... ...all 24	364 (15)
GW3965		chemical reagent	Activated	2.006	3.16E-08	↑ADRA2A,↑...all 21	445 (24)
Bay 11-7082		chemical - kinase in...	Activated	3.522	4.82E-12	↓BCL2A1,↓......all 20	257 (11)
aspirin		chemical drug	Activated	2.296	4.03E-08	↓CCL2, ↓C... ...all 19	302 (16)
Sn50 peptide		chemical toxicant	Activated	2.542	9.63E-11	↓BCL2A1,↓......all 18	409 (15)
glutamine		chemical - endogen...	Activated	2.079	1.08E-06	↓ATF4, ↓A... ...all 17	341 (19)
fingolimod		chemical drug	Activated	2.605	2.60E-07	↓BDNF, ↓C......all 16	321 (17)
Go 6976		chemical - kinase in...	Activated	2.552	9.00E-09	↓ABCA7,↓... ...all 16	279 (14)
carbon monoxide		chemical - endogen...	Activated	2.188	3.34E-08	↓ATF4, ↓B... ...all 15	339 (15)
BAPTA-AM		chemical reagent	Activated	2.189	1.21E-09	↓ATF3, ↓B... ...all 15	360 (20)
diphenyleneiodonium		chemical reagent	Activated	2.064	1.07E-06	↓CCL2, ↓C... ...all 13	337 (17)
etanercept		biologic drug	Activated	3.420	1.66E-09	↓CCL2, ↓C... ...all 12	375 (15)
apigenin		chemical - endogen...	Activated	2.964	1.40E-04	↓CCL2, ↓C... ...all 12	290 (19)
2,4,5,2',4',5'-hexachlorobiphenyl		chemical toxicant	Activated	2.309	3.06E-03	↓AGPAT4,↑...all 12	
phenylbutazone		chemical drug	Activated	2.229	8.11E-06	↑ABCC2,↑... ...all 12	241 (7)
infliximab		biologic drug	Activated	2.137	1.12E-04	↓CXCL1, ↓... ...all 11	405 (19)
resolvin D1		chemical - endogen...	Activated	2.043	1.06E-07	↓CCL2, ↓C... ...all 11	362 (15)
PS-1145		chemical - kinase in...	Activated	2.262	5.08E-11	↓CCL2, ↓C... ...all 11	296 (16)
sorafenib		chemical drug	Activated	2.720	2.81E-07	↓ATF5, ↓C... ...all 11	415 (23)

Table S4. The results of the Upstream Regulator Analysis filtered for compounds and drugs and sorted according to their predicted activity and the number of targets. For the Epi C vs Ctr treatment group, several anti-inflammatory or immunosuppressive chemicals, e.g. dexamethasone or glucocorticoid, were found to be on top of this list. All z scores are above 2 which showed they are significantly activated.

Upstream Regula...	Expr Log Ratio	Molecule Type	Predicted Activati...	Activation z-score	Flags	p-value of overlap	Target molec...	Mechanistic Net...
dexamethasone		chemical drug	Activated	5.030		9.16E-37	↑ACTG2, ↑ADA... ...all 117	198 (21)
beta-estradiol		chemical - endogenous ...	Activated	2.144		2.48E-13	↑ADAMTS1, ↑AL... ...all 75	187 (20)
dihydrotestosterone		chemical - endogenous ...	Activated	2.274	bias	1.52E-15	↑ADAMTS1, ↑A... ...all 40	214 (24)
filgrastim		biologic drug	Activated	2.990		6.56E-07	↓CD74, ↑CST3, ↓... ...all 23	
cyclosporin A		biologic drug	Activated	2.925		3.19E-06	↓AKR1B1, ↑APOD, ...all 22	139 (20)
diethylstilbestrol		chemical drug	Activated	2.302		2.30E-06	↓AQP9, ↑CCNA2, ...all 18	109 (7)
metribolone		chemical reagent	Activated	2.221	bias	1.07E-04	↑ADAMTS1, ↑A... ...all 15	223 (23)
bexarotene		chemical drug	Activated	2.699		2.52E-06	↓AKR1C1/AKR1C2 ...all 14	85 (13)
deferoxamine		chemical drug	Activated	2.280	bias	7.72E-05	↑ADGRG1, ↑CA9, ...all 12	114 (15)
cyclic AMP		chemical - endogenous ...	Activated	2.113	bias	3.12E-03	↑ADRA2A, ↓CCL2, ...all 11	168 (21)
R5020		chemical reagent	Activated	2.588	bias	5.19E-07	↑CCN2, ↑DKK1, ↑... ...all 10	102 (14)
allopurinol		chemical drug	Activated	2.530	bias	9.21E-07	↑ADAMTS1, ↓C... ...all 10	134 (16)
quercetin		chemical drug	Activated	2.138		3.91E-04	↓CCL2, ↓CXCL10, ...all 9	123 (19)
17-alpha-ethinylestradiol		chemical drug	Activated	2.157		8.17E-04	↓AQP9, ↓CD74, ↑... ...all 9	183 (19)
methapyrilene		chemical drug	Activated	2.236		1.67E-03	↑CBS/CBSL, ↑CST3, ...all 8	
GnRH-A		chemical reagent	Activated	2.425		9.62E-04	↓FST, ↑GADD45B, ...all 6	134 (12)
corticosterone		chemical - endogenous ...	Activated	2.200		1.25E-02	↑CCN2, ↑DDIT4, ↑... ...all 6	
phenylbutazone		chemical drug	Activated	2.433	bias	3.61E-04	↑ADAMTS1, ↑LA... ...all 6	
2-bromoethylamine		chemical reagent	Activated	2.425	bias	1.17E-04	↑ADAMTS1, ↑LA... ...all 6	
gentamicin C		chemical drug	Activated	2.449	bias	5.13E-04	↑ADAMTS1, ↑LA... ...all 6	
fenamic acid		chemical reagent	Activated	2.425	bias	7.68E-04	↑ADAMTS1, ↑LA... ...all 6	
triamterene		chemical drug	Activated	2.433	bias	6.05E-04	↑ADAMTS1, ↑LA... ...all 6	
vancomycin		biologic drug	Activated	2.401	bias	2.55E-03	↑ADAMTS1, ↑LA... ...all 6	
hexachlorobenzene		chemical toxicant	Activated	2.236	bias	2.03E-03	↑LAMC2, ↑MYC, ↑... ...all 5	77 (6)
lomustine		chemical drug	Activated	2.236	bias	3.38E-03	↑LAMC2, ↑MYC, ↑... ...all 5	
phenacetin		chemical drug	Activated	2.236	bias	5.01E-04	↑LAMC2, ↑MYC, ↑... ...all 5	
eicosapentenoic acid		chemical drug	Activated	2.235		1.53E-02	↓CCL2, ↓MMP8, ↓... ...all 5	
etanercept		biologic drug	Activated	2.236	bias	9.79E-05	↓CCL2, ↓CCL20, ↓... ...all 5	129 (16)
ethionine		chemical toxicant	Activated	2.000	bias	3.24E-03	↑LAMC2, ↑MYC, ↑... ...all 4	
2,4,5,2',4',5'-hexachlorobi		chemical toxicant	Activated	2.000		9.90E-02	↑AOX1, ↑EBF1, ↑... ...all 4	
